# The generation of detergent-insoluble clipped fragments from an ERAD substrate in mammalian cells

**DOI:** 10.1038/s41598-023-48769-z

**Published:** 2023-12-06

**Authors:** Grant J. Daskivich, Jeffrey L. Brodsky

**Affiliations:** A320 Langley Hall, Department of Biological Sciences, University of Pittsburgh, Pittsburgh, PA 15260 USA

**Keywords:** Biochemistry, Cell biology

## Abstract

Proteostasis ensures the proper synthesis, folding, and trafficking of proteins and is required for cellular and organellar homeostasis. This network also oversees protein quality control within the cell and prevents accumulation of aberrant proteins, which can lead to cellular dysfunction and disease. For example, protein aggregates irreversibly disrupt proteostasis and can exert gain-of-function toxic effects. Although this process has been examined in detail for cytosolic proteins, how endoplasmic reticulum (ER)-tethered, aggregation-prone proteins are handled is ill-defined. To determine how a membrane protein with a cytoplasmic aggregation-prone domain is routed for ER-associated degradation (ERAD), we analyzed a new model substrate, TM-Ubc9ts. In yeast, we previously showed that TM-Ubc9ts ERAD requires Hsp104, which is absent in higher cells. In transient and stable HEK293 cells, we now report that TM-Ubc9ts degradation is largely proteasome-dependent, especially at elevated temperatures. In contrast to yeast, clipped TM-Ubc9ts polypeptides, which are stabilized upon proteasome inhibition, accumulate and are insoluble at elevated temperatures. TM-Ubc9ts cleavage is independent of the intramembrane protease RHBDL4, which clips other classes of ERAD substrates. These studies highlight an unappreciated mechanism underlying the degradation of aggregation-prone substrates in the ER and invite further work on other proteases that contribute to ERAD.

## Introduction

Protein homeostasis, or proteostasis, is defined as the maintenance of the cellular proteome and plays a critical role in cellular and organismal health^1,2^. Although errors during transcription, protein translation, and protein folding may result in damaged proteins and compromise proteostasis, inherited or spontaneous/somatic mutations in the genome more prominently result in the production of transiently misfolded or aberrantly processed proteins. Not surprisingly, myriad diseases are linked to these events, and drugs that modulate various nodes in the proteostasis pathway are under development^3,4^. Because cells must also rapidly and dynamically adjust to environmental changes, the efficacy of inducible stress response pathways that maintain proteostasis under stressful conditions is also critical for cell and organismal health^5–7^, and drugs that modulate these pathways are also under development. One class of downstream effectors of stress response pathways is molecular chaperones, which help ensure that proteins achieve their native structures^8,9^.

Proteostasis plays a particularly critical role in the secretory pathway. As a polypeptide destined for the secretory pathway is translated and exits the ribosome, N-terminal (for soluble) or in some cases internal (for membrane proteins) hydrophobic signal sequences ultimately engage the Sec61 translocon and are either delivered into the lumen of the endoplasmic reticulum (ER) or are integrated into the ER membrane^10^. To assist protein folding, specific molecular chaperones, such as the lumenal heat shock protein-70 (Hsp70) homolog, BiP, associate with and retain nascent polypeptides in a folding-competent state^11–13^. By virtue of its ability to bind and hydrolyze ATP, BiP traps these species, but it also requires the assistance of Hsp40 homologs to identify nascent misfolded proteins, activate ATP hydrolysis, and anchor BiP to the ER membrane^14^. The release of polypeptides from Hsp70–Hsp40 cycles then requires the participation of nucleotide exchange factors (NEFs), such as GRP170 in the ER, which also bind substrates that display hydrophobic runs of amino acids and—by virtue of their nucleotide exchange activity—restore Hsp70 to the ATP-bound state^15–17^. Concomitant with this cycle, polypeptides in the ER are post-translationally modified, which facilitates protein folding and oligomeric protein assembly^18,19^.

If protein folding is delayed and misfolded proteins accumulate, an ER stress response—the unfolded protein response (UPR)—is induced, which as noted above helps maintains proteostasis^5–7^. More specifically, UPR downstream effector induce the expression of molecular chaperones, slow translation, and increase ER volume, which may rectify the potentially toxic effects of misfolded proteins. Yet another protein quality control pathway is also induced: ER associated degradation (ERAD)^20–23^. During ERAD, misfolded proteins are selected, removed (or “retrotranslocated”) from the ER, ubiquitinated, and delivered to the cytosolic proteasome for degradation. While the majority of ERAD substrates represent misfolded or aberrantly processed proteins, the ERAD pathway is also co-opted to manage levels of active ER-resident proteins^24^. How the ERAD pathway recognizes and handles its substrates compared to the vast number of nascent proteins that pass through the ER is poorly defined.

To date, our understanding of the ERAD pathway has employed both endogenous (and often disease-causing) substrates that harbor mutations, as well as artificial substrates. In one example, ERAD substrates may consist of a naturally occurring transmembrane domain (TMD) fused to a “degron”, a peptide sequence that signals degradation^25^. Degrons used experimentally are either embedded within cellular proteins or have been obtained in yeast screens (see e.g.^26–28^), and degrons derived from native, disease-associated substrates can even be aggregation-prone^29^. In this case, the ERAD pathway must somehow disaggregate the polypeptide, as the presence of aggregates might impede retrotranslocation and/or proteasome access. In a yeast model, we previously showed that integral membrane substrates with an aggregation-prone cytosolic domain require the Hsp104 molecular chaperone^29,30^, a AAA-ATPase that disaggregates and threads proteins through its central cavity^31^. The yeast homolog of p97 (Cdc48), which is also a AAA-ATPase, might additionally contribute to this process, particularly since p97 can retrotranslocate and hold some ERAD substrates in solution prior to proteasome degradation^32,33^. Alternatively, the cytosolic Hsp70–Hsp40–NEF disaggregase complex might facilitate the ERAD of aggregation-prone substrates, particularly since the complex resolves pre-formed cytosolic aggregates in higher eukaryotic cells^34^. This process can generate species competent for refolding or degradation, or in principle folding intermediates that seed aggregate formation^35,36^. To date, the resolution of ER-associated aggregates prior to delivery to the ERAD pathway is largely uncharacterized in mammalian cells.

Another impediment to protein retrotranslocation from the ER, at least for membrane proteins, is TMD hydrophobicity^37,38^. Because TMD-containing proteins are energetically lodged in the membrane—and may be combined with aggregation-prone domains—the trimming or clipping of TMDs by ER-resident proteases provides another route to kickstart retrotranslocation and degradation^39^. Through cleavage near transmembrane domains^40^, TMD-containing ERAD substrates have been shown to be clipped by the signal peptide peptidase (SPP) prior to retrotranslocation, perhaps to ease the burden on AAA-ATPases like p97 that drive extraction^41,42^ and/or to facilitate disaggregation^43^. Another protease that plays a similar role is RHBDL4^44,45^. RHBDL4 recognizes hydrophilic and positively charged residues that normally flank a transmembrane domain as well as the ubiquitin chains attached to them by E3 ligases. Only select RHBDL4 substrates have been identified, though the regulated cleavage of the OST complex by RHBDL4 was recently reported^46^. It is likely that additional ERAD substrates will ultimately be identified as substrates for RHBDL4, SPP, and other ER resident proteases^47^.

In contrast to ERAD, problematic aggregation-prone soluble proteins within the ER can alternatively be delivered for ER-phagy^48–50^, and misfolded ER proteins can even escape the ER in COPII vesicles that are then targeted for lysosome/vacuole-dependent degradation^51,52^. By using a substrate that toggles between ERAD and post-ER quality control via the multivesicular body (MVB) pathway, we showed that the ER retention and ERAD of membrane proteins in yeast correlates with both substrate aggregation^53^ and ubiquitination, thanks to the participation of an ER resident ubiquitin binding protein^54^. Nevertheless, the rules that oversee the fate of non-native membrane proteins in the ER are generally mysterious, especially in mammalian cells.

To these ends, we expressed a model substrate, known as TM-Ubc9ts, in HEK293 cells. In yeast, TM-Ubc9ts requires Hsp104 prior to ERAD-targeting. In human cell lines, we now report that TM-Ubc9ts turnover relies on both the proteasome and lysosomal proteases, but at higher temperature proteasome-dependent degradation predominates. Also in contrast to yeast, proteasome inhibition stabilizes two clipped TM-Ubc9ts products, but inhibition of p97 primarily stabilizes only the first product, consistent with a p97-requirement for partial extraction and proteasome delivery. Even though the ERAD mechanism in yeast and higher cells is generally conserved^20–23^—and analyses of disease-causing proteins in yeast are commonly recapitulated in higher cells^55^—our study highlights distinct mechanisms required for the turnover of a more problematic model ERAD substrate and suggests a pipeline to investigate the mechanisms required for other aggregation-prone substrates in human cells.

## Results

### TM-Ubc9ts undergoes ERAD in mammalian cells

**Figure 1:**
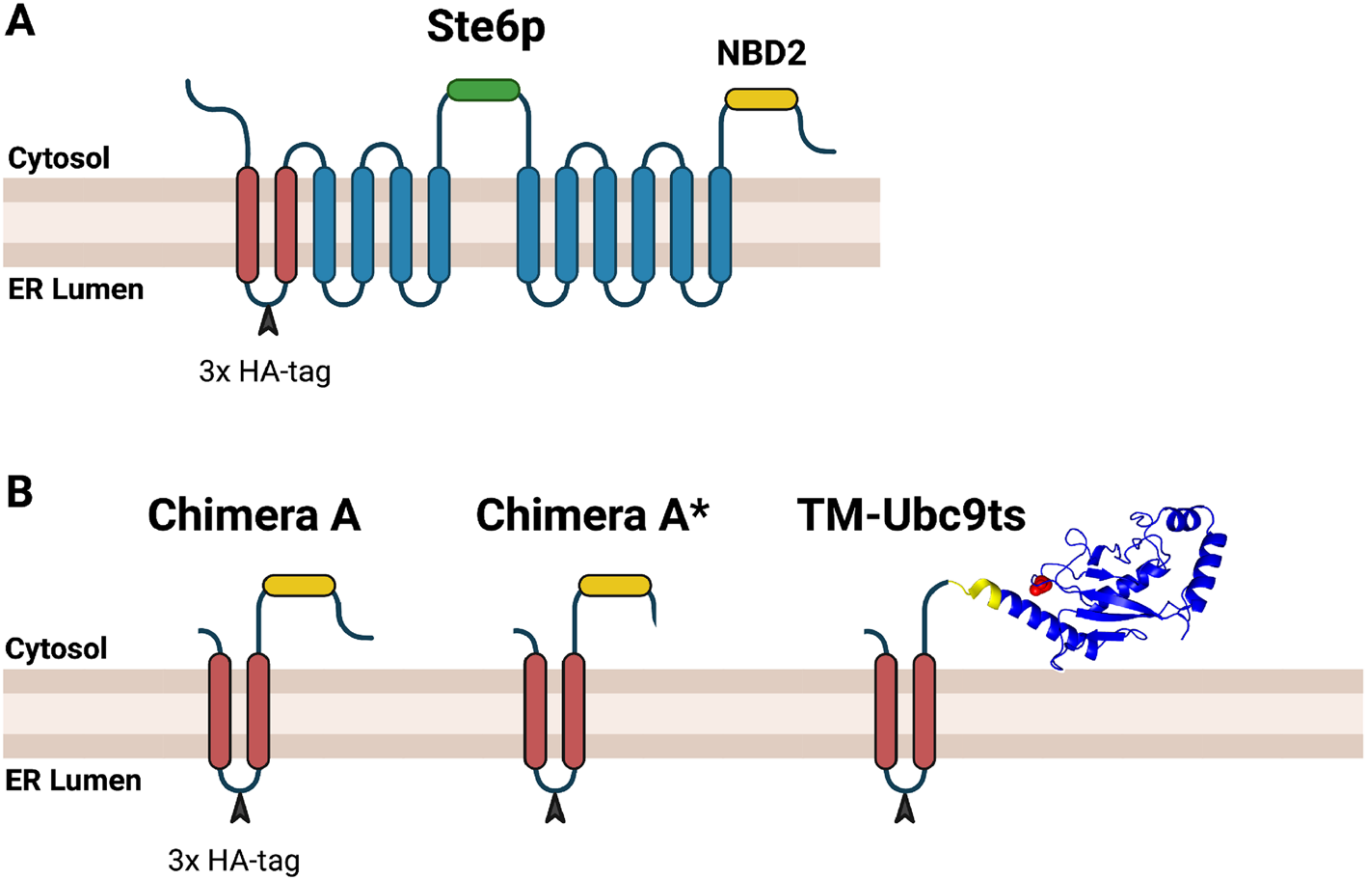
TM-Ubc9ts is a model aggregation-prone ERAD substrate. (**A**) A cartoon showing the membrane-spanning domains and the first and second nucleotide binding domain (in green and yellow, respectively) of Ste6p, a mating factor transporter in yeast. (**B**) A cartoon of the previously studied Chimera A and Chimera A* proteins, which contain a modified version of the first two membrane-spanning domains of Ste6, as well as TM-Ubc9ts, which also contains the first two transmembrane regions of Ste6p but is followed by a temperature sensitive mutant form (Y68L) of the cytosolic yeast SUMO-conjugating enzyme Ubc9. The location of Y68L in Ubc9 is depicted in red and the 3 × HA tag is marked with the black arrowhead.

To determine how an aggregation-prone ERAD substrate, TM-Ubc9ts, which requires the Hsp104 disaggregase in yeast^29^, might be handled in higher cells, we mapped the degradation pathway for this integral membrane protein. The logic underlying our project is that human cells lack Hsp104^31,56^, so we reasoned that TM-Ubc9ts might require distinct disaggregases, might be routed to a different degradation pathway (e.g., ER-phagy), or might even be stable and toxic. TM-Ubc9ts was constructed by appending the first two TMDs of Ste6, a yeast mating factor transporter^57^, to a temperature-sensitive mutant form of Ubc9^58^, a SUMO conjugating enzyme (Fig. 1). When expressed in the yeast cytosol, the Ubc9ts moiety (lacking the TMD) was sequestered into puncta dubbed “Q-bodies” after heat shock^59^. We then showed that when Ubc9ts was tethered to the ER membrane, the protein formed ER puncta, particularly after heat shock, and required Hsp104 activity for maximal proteasome-dependent degradation^29^.

To elucidate the TM-Ubc9ts degradation pathway in higher cells, we first transiently transfected HEK293 cells with an expression plasmid encoding TM-Ubc9ts and conducted cycloheximide chase assays to measure protein stability. We also performed detergent solubility assays to determine aggregation propensity. Each assay was conducted at 37 °C or after a shift to 42 °C for 1 h to investigate whether elevated temperature influences stability and/or detergent solubility, as observed in yeast. These conditions reflect an accepted method to induce temperature sensitive protein aggregation^60^.

**Figure 2:**
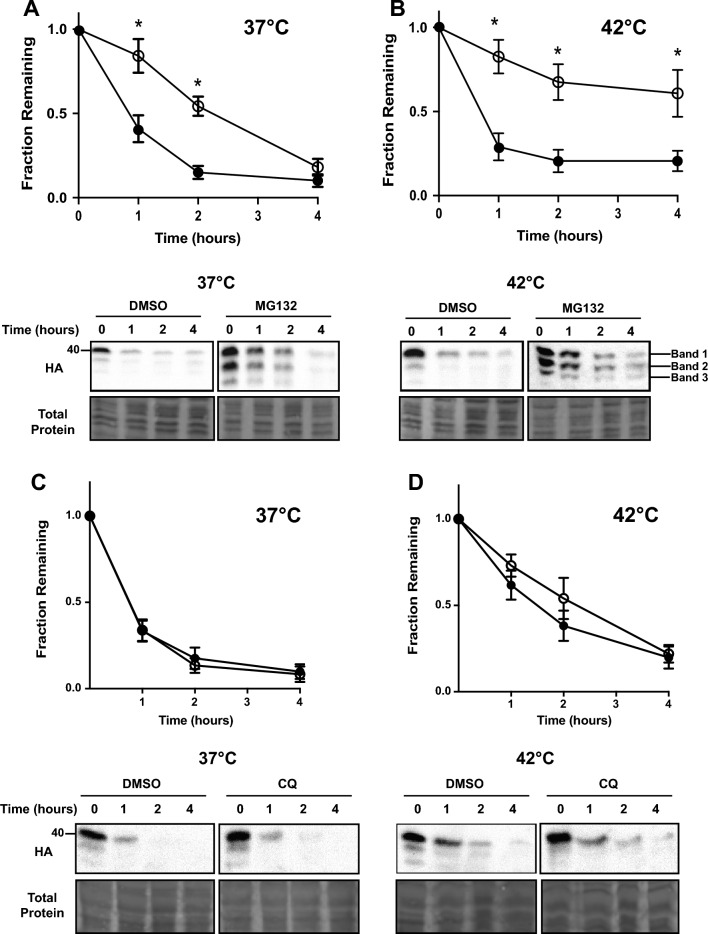
The ERAD of TM-Ubc9ts is magnified at elevated temperatures. The stability of TM-Ubc9ts was determined by cycloheximide chase analyses at 37 °C and 42 °C over 4 h. (**A**,**B**) Cells were treated with either DMSO (closed circle) or 100 μM MG132 (open circle) for the duration of the chase. (**C**,**D**) Cells were treated with either DMSO (closed circle) or 50 μM chloroquine (open circle) for 2 h prior to the chase and then throughout the chase. Western blots were visualized with anti-HA antibody to detect TM-Ubc9ts. In all panels, n = 3 independent biological experiments ± SEM; *P < 0.05. Gels are cropped from the full image.

Initially, we confirmed that TM-Ubc9ts-expressing cells exhibited robust growth, suggesting the substrate was not toxic. In fact, the substrate was efficiently degraded (Fig. 2), and in line with trends previously observed in yeast, TM-Ubc9ts was stabilized by the addition of MG132, a proteasome inhibitor^61^, even though at later times the substrate was fully degraded, at least at 37 °C (Fig. 2A). However, proteasome-dependent degradation was significantly stronger when cells were shifted to 42 °C (Fig. 2B). Moreover, at both temperatures, we observed the appearance of proteolytic fragments generated from the full-length protein (“Band 1”). This was especially apparent in the presence of MG132 (note the appearance of the “Band 2” and “Band 3” fragments), suggesting that other proteases contribute to degradation—as outlined in the Introduction—and that the proteasome might then be responsible for their degradation. Because TM-Ubc9ts clipping was absent in yeast^29^, these results indicate a distinct difference between how this substrate is recognized for ERAD in yeast versus human cell lines.

To determine if lysosome activity was required for TM-Ubc9ts turnover, especially since MG132 stabilization was incomplete at 37 °C, we conducted chases in the presence of chloroquine (Fig. 2C,D). Yet, at both temperatures, little substrate stabilization was apparent compared to the vehicle control. Therefore, the lack of more robust stabilization with MG132 at 37 °C likely reflects the impact of an alternate protease. Nevertheless, these data indicate, as shown before in yeast^29^, that the protein is primarily targeted for ERAD, but in cell culture partial proteolysis aids substrate turnover.

### TM-Ubc9ts is both more insoluble and clipped to a greater degree at higher temperatures

**Figure 3:**
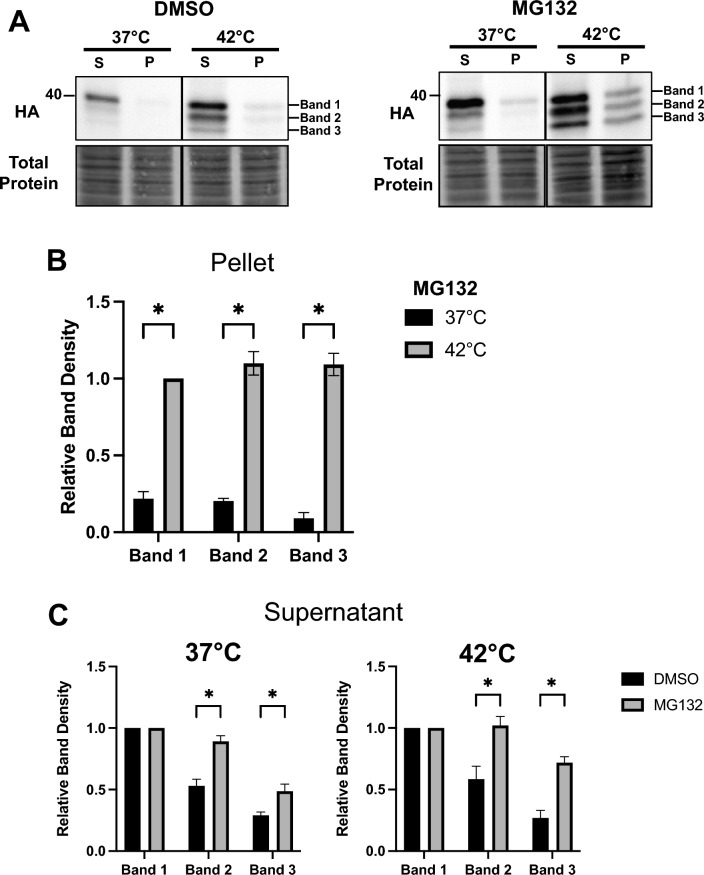
TM-Ubc9ts is clipped after incubation at elevated temperatures. Protein solubility of TM-Ubc9ts was determined in transiently transfected HEK293 cells after treatment with NP-40 to solubilize membrane proteins (soluble fraction) and then RIPA (insoluble fraction). (**A**) Levels of protein were analyzed via western blot after centrifugation and resuspension in the supernatant (S) or pellet (P) fraction. Western blots were visualized with anti-HA antibody to detect TM-Ubc9ts. (**B**) Solubility of TM-Ubc9ts was measured by comparing protein levels in the pellet fraction between DMSO and MG132 at 42 °C. All measurements were normalized to their respective band in the 42 °C pellet. (**C**) Quantification of relative band density compared to full length TM-Ubc9ts at 37 °C; all measurements were normalized to Band 1 in each respective condition, n = 3 independent biological experiments ± SEM; *P < 0.05, relative to the DMSO control. Gels are cropped from the full image.

Our prior work linked ERAD propensity to the acquisition of detergent-insolubility for select membrane proteins^29,30^. Therefore, we hypothesized that TM-Ubc9ts would become partially insoluble at 42 °C, coincident with more efficient ERAD targeting and with previous observations that cytosolic Ubc9ts aggregates after a heat shock^58,59^. Consequently, we measured TM-Ubc9ts solubility in HEK293 cells transiently transfected with the TM-Ubc9ts expression plasmid in the presence or absence of the proteasome inhibitor. For this analysis, NP-40 was used to liberate and solubilize membrane-bound proteins, based on prior work^62^. The resulting pellet was then treated with an SDS/sodium deoxycholate buffer to isolate and quantify the amount of insoluble substrate. As a control for this assay, we also examined the behavior of the F508del Cystic Fibrosis Transmembrane Conductance Regulator (CFTR), which becomes aggregation-prone at elevated temperatures^63^. As shown in Fig. S1, wild-type CFTR was more soluble than F508del CFTR at 42 °C. Next, as anticipated, TM-Ubc9ts solubility decreased in response to higher temperature incubation, especially when the proteasome was inhibited (Fig. 3A). More notable was that treatment with MG132 again stabilized the three distinct TM-Ubc9ts fragments, recapitulating results from the cycloheximide chase analyses (see above). Interestingly, the fragments primarily resided in the pellet fraction (Fig. 3B) and became more insoluble at 42 °C after MG132 treatment, which stabilized Band 2 and Band 3 (Fig. 3C). Due to the nature of detection via an HA-tag between TM1 and TM2 facing the ER lumen (Fig. 1), bands 1/2/3 are likely to include the 2 transmembrane domains as most degradation products of the C-terminus could not be monitored. It is also noteworthy that fragments generated from select soluble proteins, e.g., α-synuclein^64,65^, myoglobin^66^, and the cell cycle protein Cks1^67^, are also more aggregation-prone after cleavage. Our data suggest that a related phenomenon may be relevant during the ERAD of an aggregation-prone protein.

### The TM-Ubc9ts degradation profile in a stable expression system

**Figure 4:**
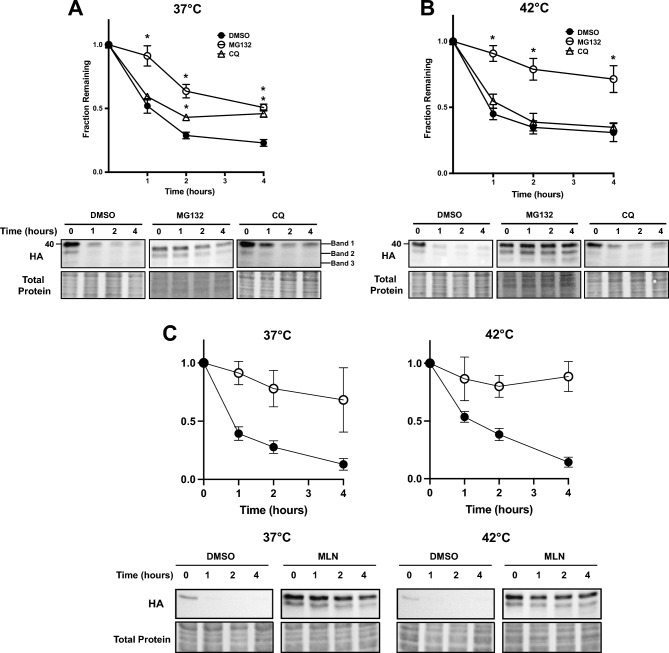
ERAD-dependent processing of TM-Ubc9ts is also temperature dependent in stable cells. The stability of TM-Ubc9ts was determined by cycloheximide chase analyses at (**A**) 37 °C and (**B**) 42 °C over 4 h. Expression was induced with 1 μg/mL tetracycline 16 h prior to the chase. Cells were treated with either DMSO (closed circle), 100 μM MG132 (open circle), or 50 μM chloroquine (open triangle) for 2 h prior to chase and then throughout the chase. (**C**) The degradation requirement for ubiquitination was determined by similar chases as shown in parts (**A**) and (**B**) in which cells were treated with either DMSO (closed circle) or 5 μM of the E1 ubiquitin activating enzyme inhibitor, MLN7243 (open circle; “MLN”). Western blots were visualized with anti-HA antibody to detect TM-Ubc9ts. In all panels, n = 3 independent biological experiments ± SEM; *P < 0.05, relative to the DMSO control. Gels are cropped from the full image.

Because transient transfection can induce secondary stress responses and expression is uncontrolled, we created stable HEK293 lines that express TM-Ubc9ts under the control of a tetracycline inducible promoter. After selecting for and isolating the inducible stable lines (see “Materials and methods”), we performed an expression time course after addition of 1 μg/mL tetracycline. TM-Ubc9ts expression was evident at 4 h and remained relatively consistent over 24 h (Fig. S2). We chose a 16 h time point for all future experiments since it ensured expression was robust, was at a level comparable to that after transient transfection and allowed for subsequent analyses. During this time course, cell growth was unchanged regardless of whether TM-Ubc9ts was expressed (data not shown). When we then performed stability assays (Fig. 4), the same overall trends as noted in the transient transfection system were observed in the presence or absence of MG132, i.e., proteasome-dependent degradation was magnified at 42 °C compared to the results at 37 °C. However, at 37 °C there was now both lysosome- and proteasome-dependent degradation, but at 42 °C ERAD again predominated. Based on the data in Fig. 4A,B, the relative magnitude of proteasome-dependent degradation (i.e., ± MG132) changes from ~ 0.25 to ~ 0.50 at 37 °C and from ~ 0.30 to ~ 0.75 at 42 °C, thus reflecting an increase in proteasome-dependence by approximately 40%. In addition and in contrast to the data in transiently transfected cells, we also noted that the proteasome and lysosomal degradation pathways were exclusively responsible for degrading TM-Ubc9ts at 37 °C (compare Figs. 2A and 4A), highlighting the value of the stable expression system. Additionally, we chose to assess TM-Ubc9ts stability when the E1 ubiquitin activating enzyme was inhibited with MLN7243 (“MLN”). As expected, TM-Ubc9ts was again strongly stabilized, confirming that ubiquitination is required for the bulk of its degradation (Fig. 4C). The distinction between the relative degree of ERAD targeting might reflect the secondary stresses triggered by transient transfection. Regardless, once again, TM-Ubc9ts-generated fragments were stabilized by MG132 treatment (see below). Coupled with data in the transient expression system, these data confirm that the degradation pathway for TM-Ubc9ts increasingly relies on the ERAD pathway at elevated temperatures.

**Figure 5:**
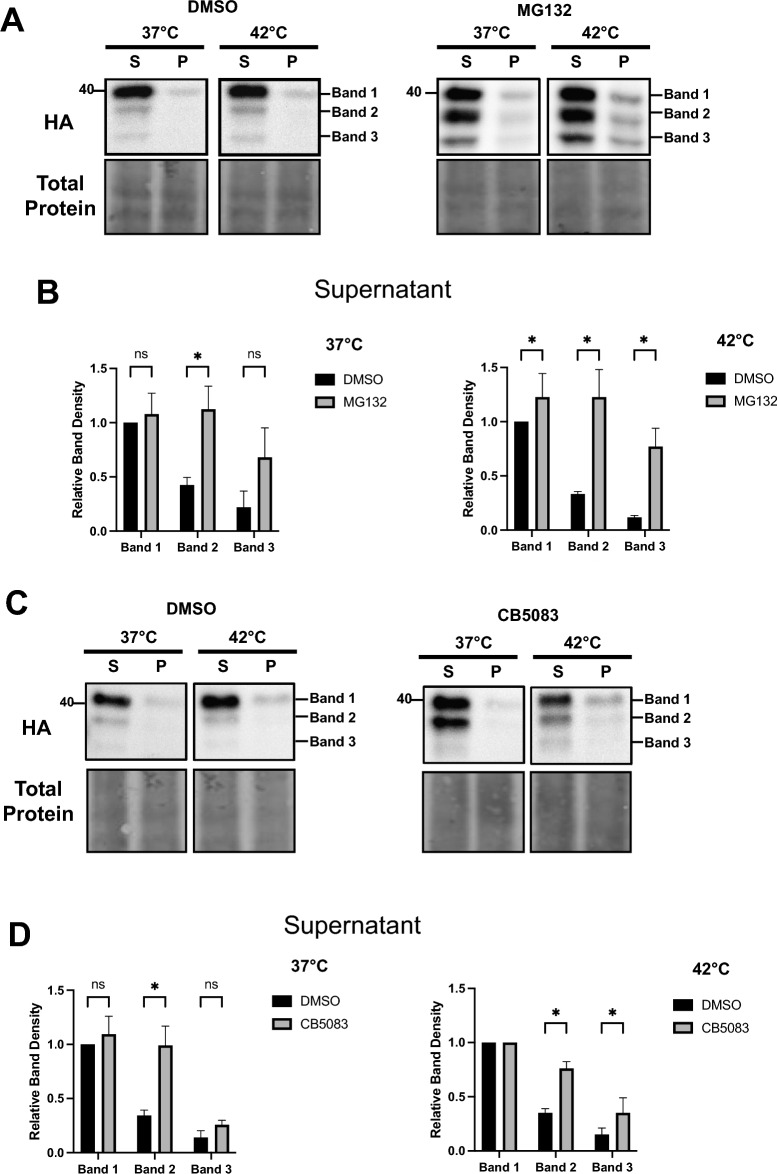
TM-Ubc9ts solubility and clipping in a stable expression system mirror results after transient transfection. Protein solubility of TM-Ubc9ts was determined in stable HEK293 cells as in Fig. 3. (**A**,**D**) Levels of protein were analyzed via western blot after centrifugation to isolate a supernatant (S) and pellet (P) fraction. Cells were treated with 100 μM MG132 (**A**,**B**) or 50 μM CB5083 (**C**,**D**) for 3 h prior to lysis. Western blots were visualized with anti-HA antibody to detect TM-Ubc9ts and chemiluminescence. Quantification of relative band density normalized to Band 1 in each respective condition; n = 3 independent biological experiments ± SEM; *P < 0.05 relative to the DMSO control. Gels are cropped from full the image.

The results presented in Fig. 4 are also consistent with data in the transient expression system that TM-Ubc9ts—and particularly the generated fragments—become detergent-insoluble. This was demonstrated by performing detergent solubility assays in the stable lines expressing TM-Ubc9ts (Fig. 5). Although protein in the insoluble (pellet) fractions was negligible after the shift to 42 °C (Fig. 5A, top, compare relative amounts in the “S” and “P” fractions at 37 and 42 °C), the aforementioned TM-Ubc9ts fragments (and to a modest extent, the full-length protein) were not only stabilized by MG132 but there was less soluble material (Fig. 5A, bottom panels, and Fig. 5B).

### TM-Ubc9ts fragments require p97 for retrotranslocation and degradation

**Figure 6:**
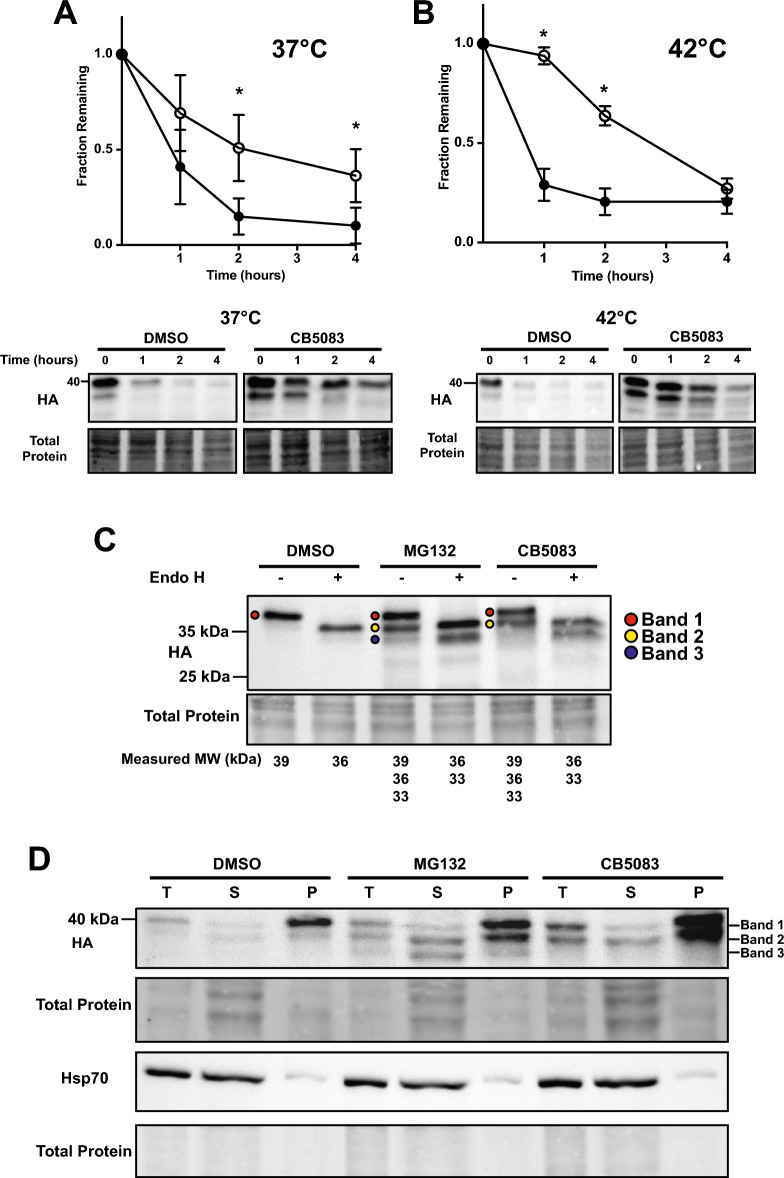
p97 inhibition liberates a proteolytic TM-Ubc9ts product. The degradation of TM-Ubc9ts expressed in stable HEK293 lines was determined by cycloheximide chase analyses at (**A**) 37 °C and (**B**) 42 °C over 4 h. Expression was induced with 1 μg/mL tetracycline 16 h prior to chase. Cells were treated with either DMSO (closed circle) or 50 μM CB5083 (open circle) for 2 h prior to chase. Western blots were visualized with anti-HA antibody to detect TM-Ubc9ts. (**C**) Endo H digestion of TM-Ubc9ts after treatment with DMSO, MG132, or CB5083. Molecular weights of the bands are listed below, rounded to two significant figures. Bands are denoted by symbols: Band 1 (red circles), Band 2 (yellow circles), Band 3 (black circles). (**D**) Carbonate extraction of TM-Ubc9ts after treatment with DMSO, MG132, or CB5083 for 3 h prior to lysis. Each treatment consists of a 1% total lysate (T), and a sample of the supernatant (S) and pellet (P) fractions. In addition to the analysis of TM-Ubc9th, cytosolic Hsp70 was examined as a control. In all panels, n = 3 independent biological experiments ± SEM; *P < 0.05. Gels are cropped from the full image.

p97 is a core component of the ATP-dependent retrotranslocation “engine”^68^, and acts on polyubiquitinated and partially trimmed ubiquitinated species at the ER membrane^69,70^. To examine if p97 also plays a role in TM-Ubc9ts degradation, we conducted another series of cycloheximide chase assays at both 37 °C and 42 °C in the presence or absence of CB5083, a p97 inhibitor that entered clinical trials^71,72^. As shown in Fig. 6A,B, TM-Ubc9ts was stabilized by CB5083, and at least at initial time points, stronger stabilization was evident at 42 °C, perhaps consistent with the magnified effect of ERAD (Fig. 4). Notably, we could detect the first clipped version of TM-Ubc9ts (“Band 2”), yet unlike the outcome that was observed with MG132, Band 3 was present at significantly lower levels (Fig. 5C). In contrast to the observed changes in the fragments after proteasome inhibition, p97 inhibition primarily impacted Band 2 stability with little to no effect on solubility (compare relative Band 2 amounts in “P” fractions, Fig. 5C,D). These data support a model in which an apparent proteasome-independent clipping at the ER membrane precedes a p97-dependent step that allows for subsequent proteasome processing (see “Discussion”). Assuming that p97 is required for retrotranslocation, the data are also in-line with results from other investigations on the action of ER proteases^39,41,46^. In our case, a protease facilitates the conversion of a full-length product into a fragment, Band 2, which is then acted upon by the proteasome.

We next sought to better characterize the two lower molecular weight species (Band 2 and Band 3) that were stabilized after MG132 treatment and migrated at molecular masses of ~ 36 kDa and ~ 33 kDa, respectively. Consequently, samples from experiments under control (DMSO) conditions or after treatment with MG132 or CB5083 were treated with endoglycosidase H (Endo H), which removes the N-glycan core from Asn-X-Ser/Thr-containing proteins in the lumen of the ER^73^. In the absence of Endo H, vehicle-treated cells express full-length TM-Ubc9ts at a molecular mass of 39 kDa, which by virtue of a single N-linked glycosylation site (Fig. 1) is trimmed to a ~ 36 kDa product by Endo H (Fig. 6C). The two processed fragments at 36 kDa and 33 kDa, which are stabilized by MG132, appear to be differentially acted upon by the enzyme. Based on the strong increase in the magnitude of the species denoted Band 3, we surmise that only Band 3 is unaffected by Endo H (since no product ~ 3 kDa lighter than this is seen), yet Band 1 and Band 2 are each acted upon by this enzyme. This modestly increases the 36 kDa species and strongly increases the levels of Band 3.

To further characterize the fragments, we treated lysates with sodium carbonate and then resolved membrane (i.e., the pellet fraction) versus soluble (i.e., the supernatant fraction) proteins after centrifugation. As depicted in Fig. 6D, Band 3, when stabilized by MG132 treatment, was almost entirely soluble, suggesting it was freed from the ER prior to degradation. In contrast, after treatment with MG132 or CB5083, Band 2 remained mostly membrane integrated (pellet) but a more minor fraction resided in the supernatant. As a control for fractionation, the behavior of Hsp70, which is largely cytosolic, was also followed and resided primarily in the supernatant fraction. Combined with the data from experiments with Endo H (Fig. 6C), we hypothesize that Band 2 is primarily retrotranslocated by p97 after being clipped. The presence of the minor population of Band 2 in the supernatant, even after p97 was inhibited (Fig. 6C, CB5083, “S”), suggests that the proteasome retrotranslocates Band 2^74–76^, albeit inefficiently. When combined with data on the relative effects of MG132 and CB5083, we present a model for the TM-Ubc9ts degradation pathway in the “Discussion” section, below.

### The intramembrane protease RHBDL4 does not cleave TM-Ubc9ts

**Figure 7:**
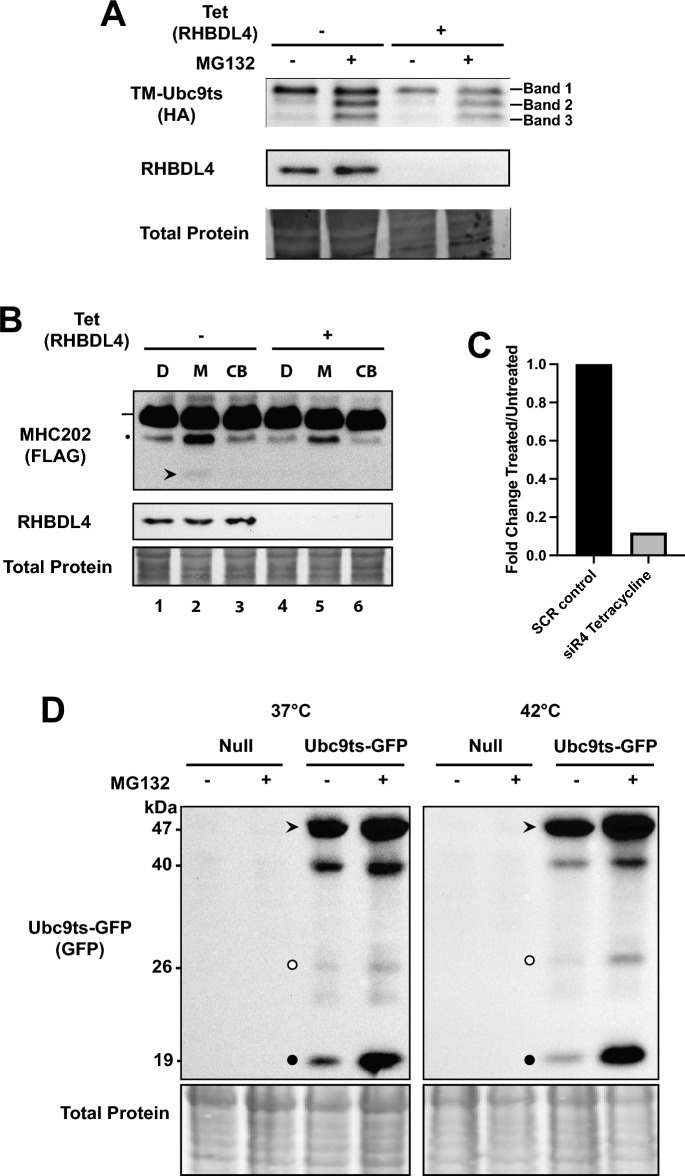
TM-Ubc9ts cleavage is RHBDL4-independent. (**A**) TM-Ubc9ts was transiently transfected into HEK293 cells 72 h after tetracycline induction of an shRNA to deplete RHBDL4, i.e., the addition of tetracycline (“Tet”) leads to shRNA expression and the loss of RHBDL4. Under both DMSO and MG132 treated conditions, full-length substrate (Band 1) and TM-Ubc9ts fragment (Bands 2 and 3) abundance and ratio were unchanged between control and knockdown conditions. (**B**) MHC202 was transiently transfected into HEK293 cells 72 h after tetracycline induction of an shRNA against RHBDL4: “D”, DMSO; “M”, MG132; “CB”, CB5083. Full-length MHC202 is indicated by a line, and deglycosylated MHC202 is denoted by a closed circle (•). Note that the cleaved fragment (arrowhead) stabilized by MG132 in lane 2 (− tet) was absent in lane 5 (+ tet). (**C**) qPCR quantification of RHBDL4 mRNA levels under shRNA knockdown conditions used in (**A**) and (**B**). (**D**) HEK293 cells transiently transfected with the cytosolic quality control substrate, Ubc9ts-GFP, were incubated at 37 °C or were shifted to 42° and treated with DMSO or MG132 for 1 h prior to harvesting and cell lysis. Full-length protein (arrowhead), GFP (open circle), and a cleaved fragment (closed circle) are indicated. Note the increase in the amount of the cleaved fragment in the presence of MG132 and at 42 °C. Gels are cropped from the full image.

Because a TM-Ubc9ts-generated species (i.e., Band 2) is acted upon by p97 and the proteasome, we asked if an intramembrane protease, RHBDL4, which exhibits similar features and can clip select ERAD substrates and acts prior to retrotranslocation^45,77^, contributes to TM-Ubc9ts turnover. Therefore, we utilized a stable HEK293 cell line with tetracycline-inducible expression of an RHBDL4-directed shRNA. Real-time qPCR analysis of tetracycline- versus vehicle-treated cells and western blot analysis indicated that RHBDL4 message was depleted by ~ 90%, and the protein levels were similarly reduced such that < 10% of the protein remained (Fig. 7A). When we then introduced TM-Ubc9ts into the treated versus untreated cells, the density of the three bands corresponding to TM-Ubc9ts was unchanged, regardless of whether RHBDL4 was knocked-down and whether MG132 was present (Fig. 7A). RHBDL4 knockdown was verified by RT-qPCR, and consistent with the immunoblot data in Fig. 7B, RHBDL4 mRNA was present at < 10% in the knockdown condition compared to the scrambled shRNA control (Fig. 7C). As a control for this experiment, previously studies established that this level of RHBDL4 reduction was sufficient to stabilize a C-terminal fragment derived from a model ERAD substrate, known as MHC202^45^. Therefore, we expressed MHC202 in the same system and observed the formation of an MG132-stabilized cleaved fragment at ~ 15 kDa (Fig. 7B, see arrowhead, lane 2), consistent with previous studies. However, the fragment was absent when RHBDL4 was knocked-down (Fig. 7B, see lane 5). The lack of an effect of RHBDL4 on TM-Ubc9ts cleavage, as seen in our hands, is perhaps consistent with the fact that TM-Ubc9ts contains a misfolded domain that faces the cytosol. In contrast, established RHBDL4 substrates, such as pTα, Pkd1ΔN, Opsin-degron, MPZ-L170R, TCRα are cleaved primarily in juxtamembrane and loop regions^77^.

The data presented above, along with the residence of the Ubc9ts misfolded domain, suggest instead that cleavage might require a cytosolic (and not an ER resident) protease. To test this hypothesis, we examined a soluble form of TM-Ubc9ts that lacks the TMD. This cytosolic quality control substrate, Ubc9ts-GFP^59^, was then introduced into HEK293 cells. We also incubated cells at the aforementioned temperatures (37 °C or 42 °C for 1 h prior to lysis) and in the presence or absence of MG132. As shown in Fig. 7D, the full-length protein (~ 47 kDa, arrowhead), GFP (~ 26 kDa, open circle), as well as several fragments (see, for example, the closed circle at ~ 19 kDa) were each observed. Interestingly, MG132 treatment strongly suggested that ~ 26 and 19 kDa fragments are turned over by the proteasome, especially at 42 °C, given their increase in intensity. Because both TM-Ubc9ts and Ubc9ts-GFP share the same temperature-sensitive Ubc9 domain, these data suggest that a cytosolic protease cleaves both the ER-tethered and cytosolic model substrates.

## Discussion

**Figure 8:**
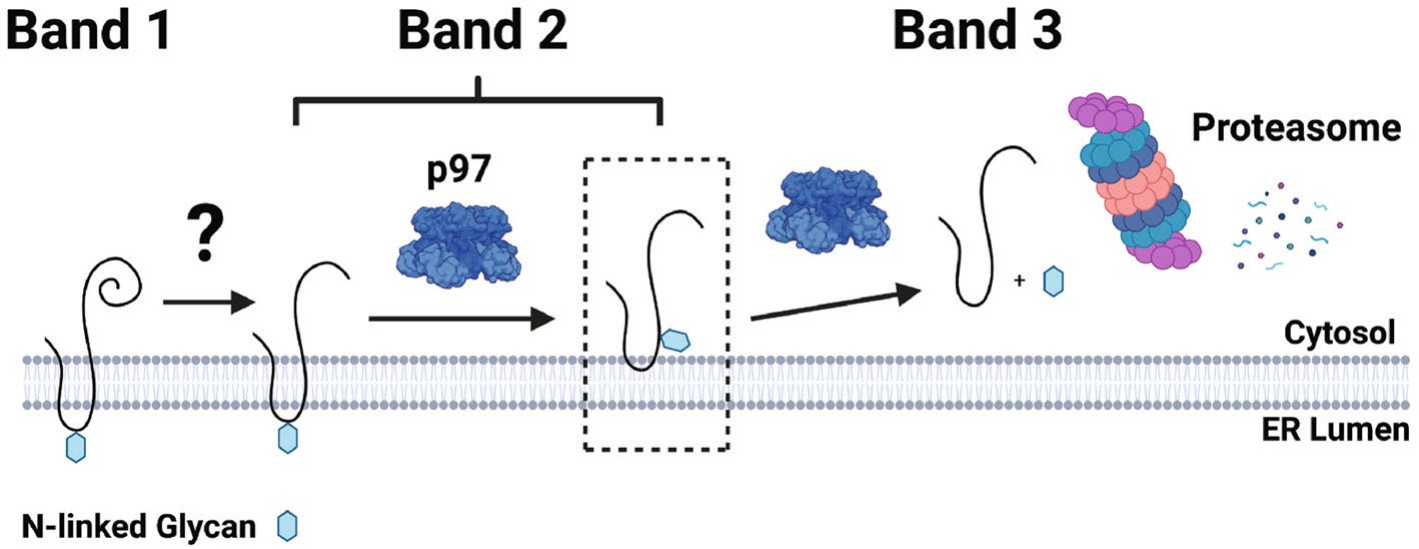
Proposed model for TM-Ubc9ts clipping during ERAD. TM-Ubc9ts is shown as a solid black line in the ER membrane with its sole N-linked glycan depicted as a hexagon. As TM-Ubc9ts is processed by ERAD, it adopts three states, labeled in this figure as Band 1 (full length), Band 2 (clipped and glycosylated), and Band 3 (clipped and deglycosylated). Full-length TM-Ubc9ts is clipped prior to retrotranslocation by a cytosolic protease in the transition from Band 1 to Band 2. Clipped TM-Ubc9ts is then extracted from the ER membrane by p97 in preparation for degradation. It is during this step that two populations of Band 2 can be observed via carbonate extraction, with the majority residing in the ER membrane as well as a small pool in the cytosol en route to the proteasome. Band 3 represents clipped TM-Ubc9ts that has been deglycosylated by glycosidases in the cytosol before ultimately being degraded by the proteasome.

While substantial work from many labs has revealed how misfolded proteins are targeted and processed by ERAD, significant details of how TMD-containing aggregation-prone substrates are degraded are lacking. Our results show that one such substrate, TM-Ubc9ts, is clipped in HEK293 cells after being transiently or inducibly expressed, and that the resulting fragments can aggregate, especially if stabilized by proteasome inhibition and if cells are incubated at higher temperatures. In other words, a human cell line overcomes the lack of the Hsp104 disaggregase by clipping an aggregation-prone protein in the ER membrane. In addition, while one generated fragment, Band 2, appears to retain the N-linked glycan, Band 3 lacks this post-translational modification. In contrast, only the glycosylated and truncated species, Band 2, is primarily stabilized when p97 is inhibited. Consistent with the action of the p97 complex^37, 68^, this species remains mostly membrane-integrated (Fig. 6D). Based on these data, we present a model for the degradation pathway of TM-Ubc9ts in human cells (Fig. 8).

A growing number of ER-associated proteases contribute to the ERAD pathway^39,78–80^. While a comprehensive analysis of the contributions of each protease, which may even act redundantly, is beyond the scope of the current study, we could rule out the possibility that the intramembrane protease RHBDL4 is responsible to generate these fragments. Yet, it is noteworthy that other proteases act more specifically on substrates based on the frequency and composition of TMDs or based on the site of lesions or recognition motifs. For example, presenilin, which is part of the gamma-secretase complex and several members of the rhomboid protease family initiate biological pathways through the clipping of substrates such as amyloid precursor protein (APP), the Notch receptor, and receptor tyrosine kinases^41,81,82^. More specifically, gamma secretase is required to cleave Notch receptors as well as distinct receptor tyrosine kinases, whereas APP is cleaved by both alpha and gamma secretase during non-amyloidgenic processing^83–85^. While these substrates lack homology, they all possess cleavage sites close to the membrane. After cleavage, functional motifs bound for cellular compartments, such as the nucleus or the extracellular environment, are liberated. In contrast, RHBDL4 primarily acts on ubiquitinated proteins at the ER membrane that must be prepared for retrotranslocation and proteasome delivery.

To better dissect the requirements for the ERAD of TM-Ubc9ts in human cells, future experiments will identify partners—and ideally the contributing protease—through the use of activated crosslinking (“proximity labeling”) methods, such as BioID^86,87^. For instance, TM-Ubc9ts could be appended to BirA*, a promiscuous biotin ligase, and after pull-down with streptavidin TM-Ubc9ts partners might be identified by mass spectrometry. A translationally encoded tetra-ubiquitin at the N-terminus could also be added to TM-Ubc9ts to compare how the protein interactome changes. Because RHBDL4 has both a substrate and ubiquitin-interacting domain^45^, it stands to reason that other intramembrane proteases might only cleave proteins marked for degradation by a polyubiquitin moiety. Regardless, because the cytosolic Ubc9ts-GFP substrate is also cleaved, and the fragments are stabilized when cells are incubated with a proteasome inhibitor, we suggest that a cytosolic protease is responsible for TM-Ubc9ts clipping. Mapping the precise cleavage site after fragment purification by mass spectroscopy—along with the use of programs that predict protease dependence^88^—highlights another route to identify the protease.

One potential limitation of a comparison between the behavior of TM-Ubc9ts in yeast and HEK293 cells is that experiments were performed at 30 °C and 37 °C in yeast, but 37 °C and 42 °C in HEK293 cells. Thus, formally, substrate clipping in yeast might have only arisen at the higher temperature of 42 °C. Nevertheless, an examination of TM-Ubc9ts has uncovered a previously overlooked step in the quality control of an ERAD substrate containing an aggregation-prone domain in the cytosol.

Although we have focused on a single model substrate in this study, it is likely that mutated versions of naturally occurring substrates that exhibit similar features as Ubc9ts meet the same fate. Other aggregation-prone and likely misfolded ER membrane proteins have been reported in the literature. Rhodopsin mutants, some of which are aggregation-prone in model systems (e.g., P23H), were variably targeted for ERAD or an ER-phagy-like pathway when overexpressed^89–91^. P23H rhodopsin aggregation causes retinitis pigmentosa and retinal degeneration, while wild-type rhodopsin is readily degraded by ERAD. During rhodopsin processing, we hypothesize that clipping via an intermembrane protease, such as RHBDL4, could facilitate retrotranslocation and proteasome-dependent degradation. Intriguingly, P23H rhodopsin was clipped after it was expressed in a *Xenopus laevis* model for retinitis pigmentosa^92^. In the future, introducing wild-type versus the P23H mutant rhodopsin into a higher cell systems in which RHBDL4 and other putative proteases could be silenced, and then examining detergent solubility and the generation of fragments, might allow one to group TM-Ubc9ts with this substrate.

Another example is provided by a mutant aquaporin, AQP2-T126M, which is CHAPS-resistant in CHO cells and retained in the ER in transfected cells and in mice, but the substrate can also be targeted for ERAD^93–95^. In turn, we previously showed that the N1303K cystic fibrosis transmembrane conductance regulator (CFTR) mutant is polyubiquitinated ~ 3 × more than wild-type CFTR in a reconstituted system, yet in transfected cells the protein was detergent soluble, appeared to be ERAD-resistant, and was destroyed via an autophagy like mechanism^96^. Other work reported that N1303K and different CFTR mutants reside in cytoplasmic aggresomes, which can be turned over by autophagy^97^. Considering the plethora of substrates and proteases yet to be characterized, our analysis of TM-Ubc9ts quality control provides the groundwork for significant continued work and contributes to our understanding of how aggregation prone proteins in the ER might potentially be handled in higher cells.

## Materials and methods

### Molecular methods

The expression of the TM-Ubc9ts substrate was first reported by Preston et al.^29^, and contains a temperature sensitive mutant form of the SUMO-conjugating enzyme Ubc9, which results in a temperature sensitive folding defect^58^, fused to the sequences encoding the first two transmembrane (TM) domains of the yeast ABC transporter Ste6. A triple HA tag was also inserted between the first and second transmembrane domain, which resides within the ER lumen after the protein inserts in the ER membrane^38^ (see Fig. 1). The resulting fusion protein was then inserted into pcDNA3.1 at the EcoRI and XhoI sites and amplified using 5-α Competent *E. coli* cells (NEB). The plasmid DNA sequence was confirmed by Plasmidsaurus and the insert was confirmed by primer-directed sequencing (Genewiz).

### Human cell culture protocols

HEK293H cells (here referred to as HEK293 cells) were grown in a 37 °C humidified incubator supplemented with 5% CO_2_ in DMEM containing 10% FBS as well as penicillin and streptomycin. All experiments were conducted with cells before passage four. HEK293 cells were transfected at 50% confluency with the indicated plasmids using Lipofectamine 2000 (Thermofisher). Total transfected DNA was constant for each experiment at 2 μg/well of a 6-well plate or 1 μg/well of a 12 well plate. Cells were harvested 24 h post transfection unless indicated otherwise. For all experiments, cells were collected by pipetting and centrifuged to obtain a cell pellet. The media was then aspirated, and the pellet was retained.

To isolate stable, tetracycline-inducible HEK293 cells expressing TM-Ubc9ts, we used the T-Rex inducible protein expression system (Thermofisher). In brief, HEK293 cells were first transfected with plasmid pcDNA6/TR and then selected in blasticidin as per the manufacturer’s specification. Lines selected for stable integration of pcDNA6/TR were next transfected with an inducible expression vector containing the TM-Ubc9ts insert and selected with zeocin. The final selection of single cells that induced substrate expression occurred via dilution after several 2–3 day intervals (2 weeks total) of media changes in the presence of zeocin.

### siRNA-mediated knockdown and RT-qPCR

siRNA knockdown using oligonucleotides was conducted according to the manufacturer’s protocols (Horizon; but also see below). Inhibition of the proteasome, p97, or lysosomal proteases was performed by the addition of MG132, CB-5083 (hereafter, "CB5083"), or chloroquine (2 h, 2 h, and 4 h, respectively; see below). DMSO was used as a vehicle control for all drug treatments, and apart from solubility analyses (see below), cell lysis was conducted in RIPA buffer (50 mM HEPES, pH 7.4, 150 mM NaCl, 0.1% sodium dodecyl sulfate, 0.5% sodium deoxycholate) after cells had been washed with PBS. Prior to analysis, the mixture was centrifuged for 10 min at 12,000 rcf (4 °C) to remove insoluble material.

siRNA mediated knockdown of RHBDL4 was instead conducted in HEK293 Flp-In T-REx cells (Invitrogen) with inducible expression of an shRNA against RHBDL4 (a kind gift from the Lemberg Lab^77^). Cells were plated to 20% confluency and then cultured in media containing 1 μg/mL tetracycline for the indicated times, ranging from 3 to 5 days. Prior to collection at these times, cells were transfected with the pcDNA3.1 transient expression vector for TM-Ubc9ts for 16 h prior to harvesting.

To quantify the extent of siRHBDL4 knockdown by siRNA transfection or inducible expression of shRNA (see above), total cellular RNA was extracted from HEK293 cells using the RNeasy mini kit (Qiagen) at the indicated time point. Extraction included a DNaseI digestion step as per protocol. The qScript cDNA Superscript Mix (Quantabio) was used for subsequent cDNA synthesis and conducted under the following PCR conditions: 5 min at 25 °C, 30 min at 42 °C, and 5 min at 85 °C, and then storage at 4 °C until further analysis. Each real-time (RT) qPCR reaction contained 80 ng cDNA, forward and reverse primer (forward: 5′-GGGTCGAACTTGTGGCTATT-3′; reverse: 5′-GAGGCCCTTGAGTGTACATTAG-3′) mix, and a SybrGreen I Mastermix (ThermoFisher Scientific). Each reaction was conducted using the Applied Biosystems 7300 RT PCR system at the following thermal cycling conditions: 10 min at 95 °C, 40 cycles of three steps including 15 s at 95 °C, 1 min at 60 °C, and 30 s at 95 °C. The C_T_ levels in each experiment were normalized to actin, and the Δ-C_T_ was calculated as C_T RHBDL4_–C_T actin_ for each time point and normalized to the 0 min time point.

### Protein solubility assays

HEK293 cells were plated at ~ 50% confluency and grown for 24 h before transfection or TM-Ubc9ts induction. Transient transfection (2 μg DNA per well) and tetracycline induction (1 μg/mL) were both conducted 16 h prior to harvest. Cells were either kept at 37 °C or shifted to 42 °C for 1 h to prior to harvesting. To harvest cells, they were dislodged from plates using a steady stream of media, harvested by centrifugation, and the supernatant was aspirated. The pellets were next resuspended in 150 μL of ice-cold NP40 Buffer (50 mM HEPES, pH 7.4, 150 mM NaCl, 1% NP40) and incubated on ice for 30 min. The solution was then centrifuged for 10 min at 12,000 rcf (4 °C) and the supernatant was aspirated and retained (i.e., the soluble NP40 fraction). The pellets were then resuspended in RIPA Buffer (50 mM HEPES, pH 7.4, 150 mM NaCl, 0.1% sodium dodecyl sulfate, 0.5% sodium deoxycholate), with 1 U/mL of benzonase added, and incubated end-over-end at 4 °C for 1 h or overnight, as indicated, to obtain the pellet (insoluble) fraction.

To analyze the extracted proteins via SDS-PAGE, 50 μL of sample buffer with fresh 5% β-mercaptoethanol was added to 150 μL of each sample. After incubation at 37 °C for 30 min, 15 μL of each sample was loaded onto 12.5% SDS polyacrylamide gels. Proteins were then transferred onto nitrocellulose membranes and stained with Revert 700 Total Protein Stain (Li-Cor) and imaged at 700 nm to detect total protein. After reversing the Revert Total Protein stain and rinsing in double distilled water, the blots were blocked in 1% milk/TBST for 1 h before incubation with anti-HA HRP antibody (Roche Diagnostics) or anti-Hsp70 antibody (StressMarq Biosciences) overnight at 4 °C. The blots were next washed in TBST prior to treatment with SuperSignal Femto Substrate (Thermofisher) and imaged using Bio-Rad Gel Doc equipment and software. Blots were quantified in ImageJ. Approximately equal sample loading was confirmed in all experiments via the analysis of the Revert Total Protein Stain, as shown. Loading controls include both those that were probed on the same gel in which specific images are shown or were from the same samples that were examined later. Differences in contrast between individual controls results from slight variations in the stain incubation and washes.

### Protein stability assays

HEK293 cells were grown to 50% confluency and then transfected with plasmids engineered to express TM-Ubc9ts or treated with 1 μg/mL tetracycline to express TM-Ubc9ts in the stable lines (see above). After incubation for 24 h at 37 °C for transfected cells or 16 h after induction in stable cells, 200 μg/mL of cycloheximide was added to each well. To measure proteasome-dependent degradation, MG132 at a final concentration of 50 μg/mL was added 2 h prior to the chase to half of the wells, and an equal volume of DMSO was added to the other (control) wells. To measure lysosome-dependent degradation, 50 μM chloroquine (Sigma-Aldrich; final concentration) or DMSO was added 2 h prior to chase. To inhibit substrate ubiquitination, 5 μM MLN7243 (Selleck Chemicals) or DMSO was added 2 h prior to chase. To inhibit p97, 10 μM CB5083 (Selleck Chemicals) or DMSO was added 2 h prior to chase. Plates were incubated at either 37 °C or 42 °C, and samples were collected as above, except that a Protease Inhibitor Cocktail Tablet (Roche Diagnostics) was included during the resuspension of the samples prior to SDS-PAGE/western blot analysis, as indicated above.

### Carbonate extraction

HEK293 cells were plated and grown to ~ 50% confluency before induction of TM-Ubc9ts expression 16 h before lysis (see above). As above, the cells were treated with DMSO, MG132, or CB5083 for 3 h prior to lysis. Next, the HEK293 cells were harvested as above and pellets were collected by centrifugation at 800*g* for 10 min at 4 °C followed by resuspension in 150 μL cold 10 mM Tris–HCl, pH 7.5, with protease inhibitors. Lysis was performed using needle passage before being centrifuged once again, as above. The supernatant was collected and then spun at 100,000*g* for 1 h at 4 °C to isolate a membrane pellet, which was resuspended in 150 μL RIPA lysis buffer. Next, 15 μL was set aside as the “total protein” while the remaining lysate was split and treated with either ice-cold 0.2 M sodium carbonate, pH 12, or 10 mM Tris–HCl, pH 7.5. Samples were incubated for 30 min on ice before another spin at 100,000*g* for 1 h at 4 °C. At this step, the supernatants were collected as the soluble fraction while pellets were resuspended as the membrane fraction. All samples were brought to pH 7.5 with acetic acid before adjusting samples to equal volumes and SDS-PAGE analysis.

### Statistical methods

For all statistical analyses, a one tailed Student’s *t*-test with unequal variance was done with p value < 0.05 for significance. All experiments were done with at least three biological replicates, as indicated in the figure legends. The equation used was:

$$t = x - \mu\, s/\sqrt{n}$$

where *x* = mean, μ = population mean, s = standard deviation, n = sample size.

### Supplementary Information

Supplementary Figures.

## Data Availability

The datasets generated and/or analyzed during the current study are available from the corresponding authors on reasonable request.
