# Supplementary material for: The generation of detergent-insoluble clipped fragments from an ERAD substrate in mammalian cells
Source: Sci Rep. 2023 Dec 6;13:21508. doi: 10.1038/s41598-023-48769-z (PMC10700608; doi:10.1038/s41598-023-48769-z)
Supplement: Supplementary file 1 — Supplementary Figures. [file 41598_2023_48769_MOESM1_ESM.pdf]

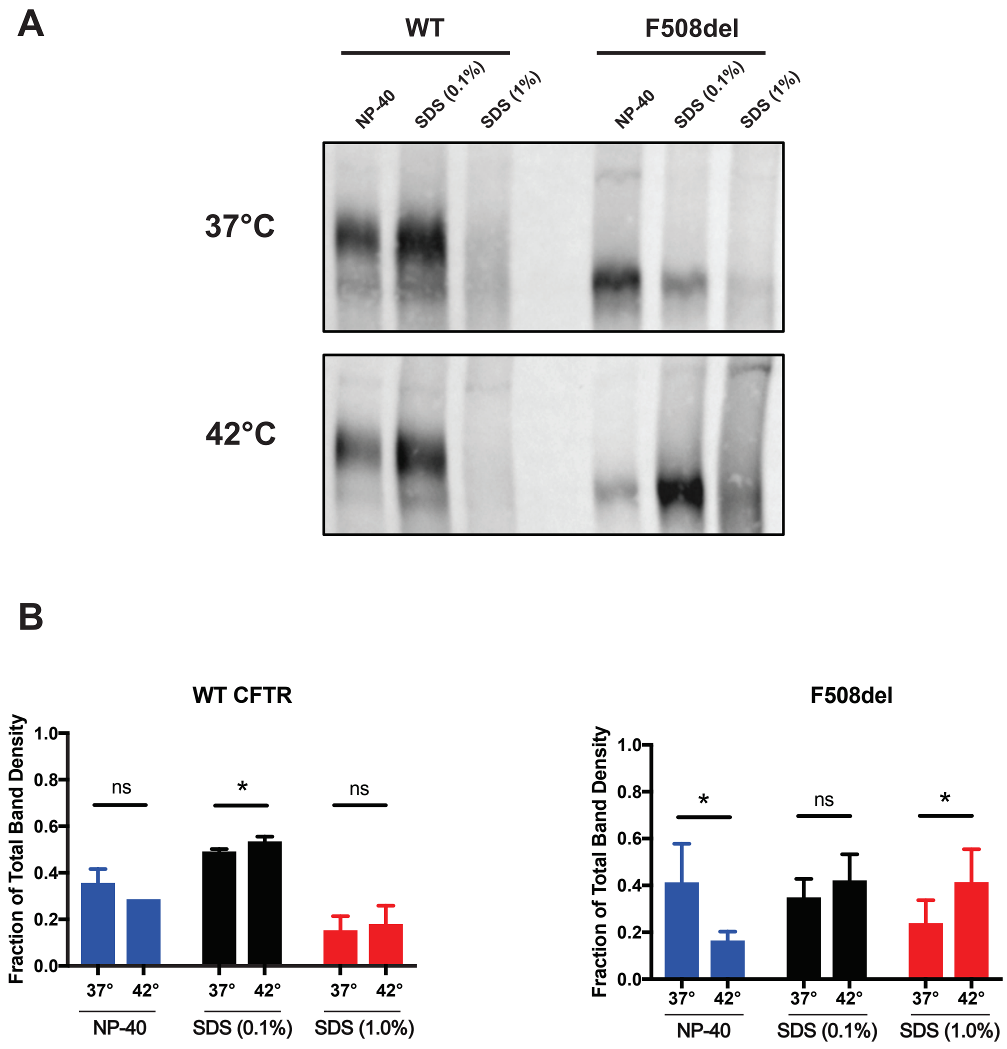

Figure S1. (A) HEK293 cells expressing either wild type or F508del CFTR at 37°C or after a 1 hour shift to 42°C were sequentially lysed and pelleted in the labeled detergents. (B) Quantification of the data in panel A; n=4, * = $p<0.05$

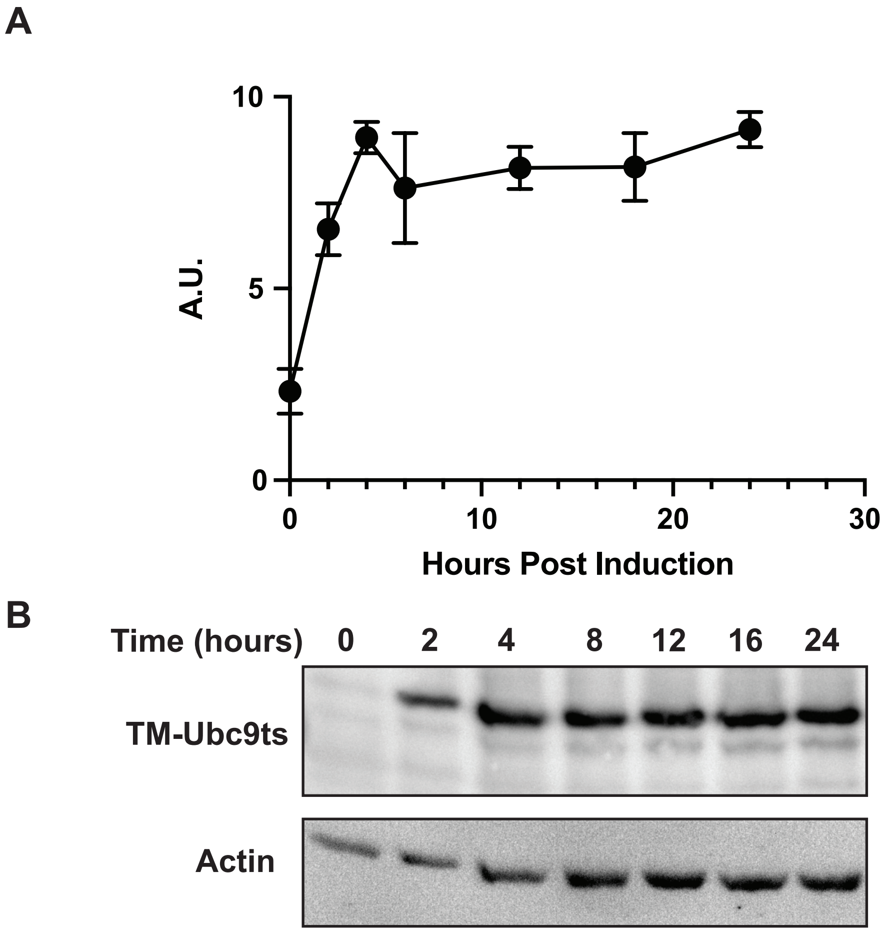

Figure S2. Time dependent induction of TM-Ubc9ts in a tetracycline-inducible expression system in HEK293 cells. A total of 1μg/ml tetracycline was added for the designated time prior to cell lysis and western blot. (A) Protein levels were normalized to actin; n = 3, +/- SEM. (B) A representative western blot of the time course is shown.

Figure S3. The following pages depict the full blots and exposures from which segments were cropped to be used in the indicated main figures in this paper. In each panel, a box indicates the cropped portion used in the corresponding figure. Loading controls include both those that were probed on the same gel in which specific images are shown or were from the same samples that were examined later.

Figure 2: anti-HA (TM-Ubc9ts)

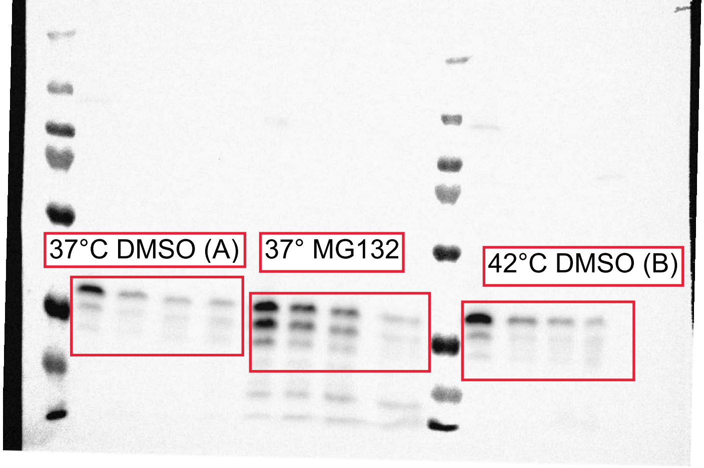

Figure 2: anti-HA (TM-Ubc9ts)

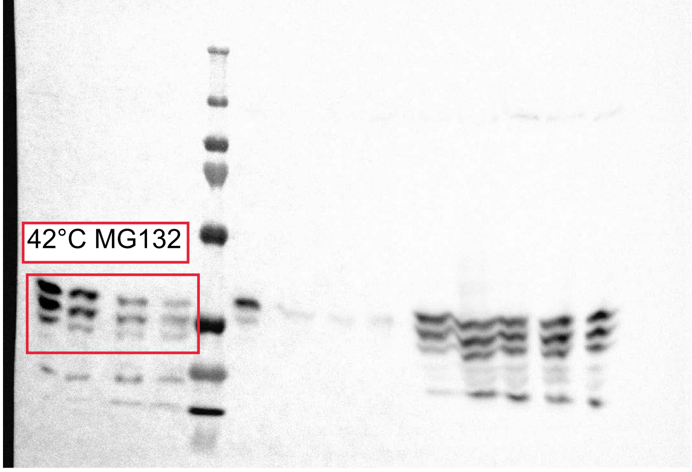

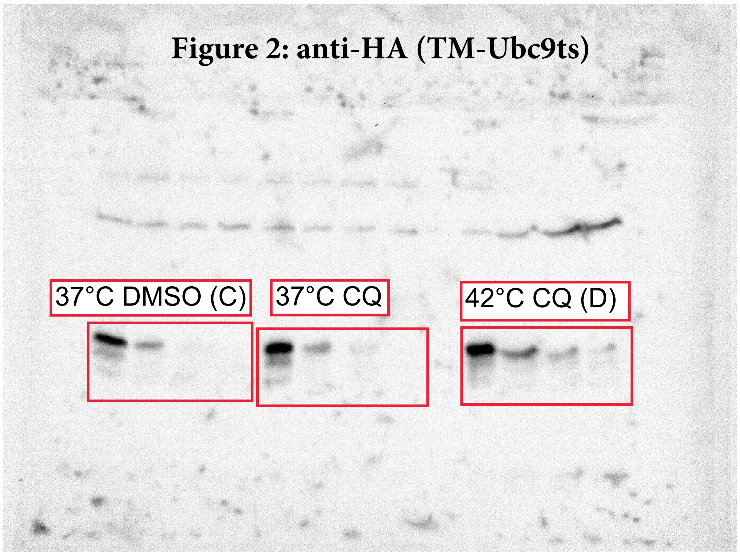
Figure 2: anti-HA (TM-Ubc9ts)
37°C DMSO (C)
37°C CQ
42°C CQ (D)

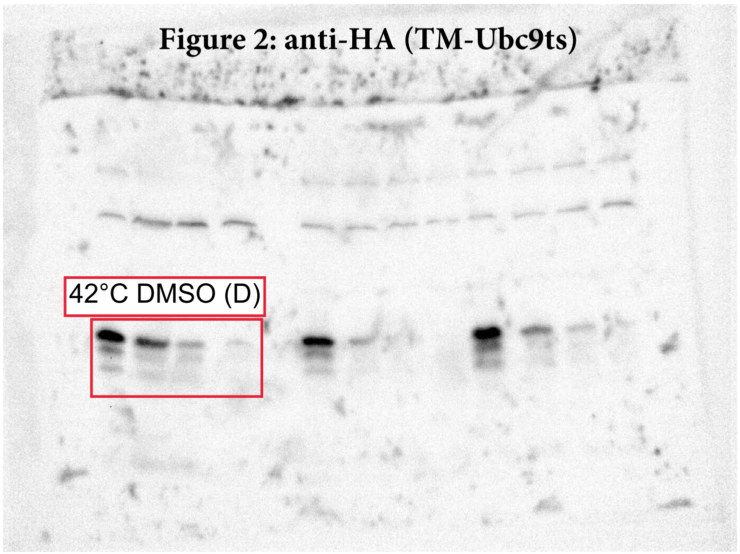

Figure 2: anti-HA (TM-Ubc9ts)

Figure 2
37 and 42°C DMSO and MG132
(A,B)

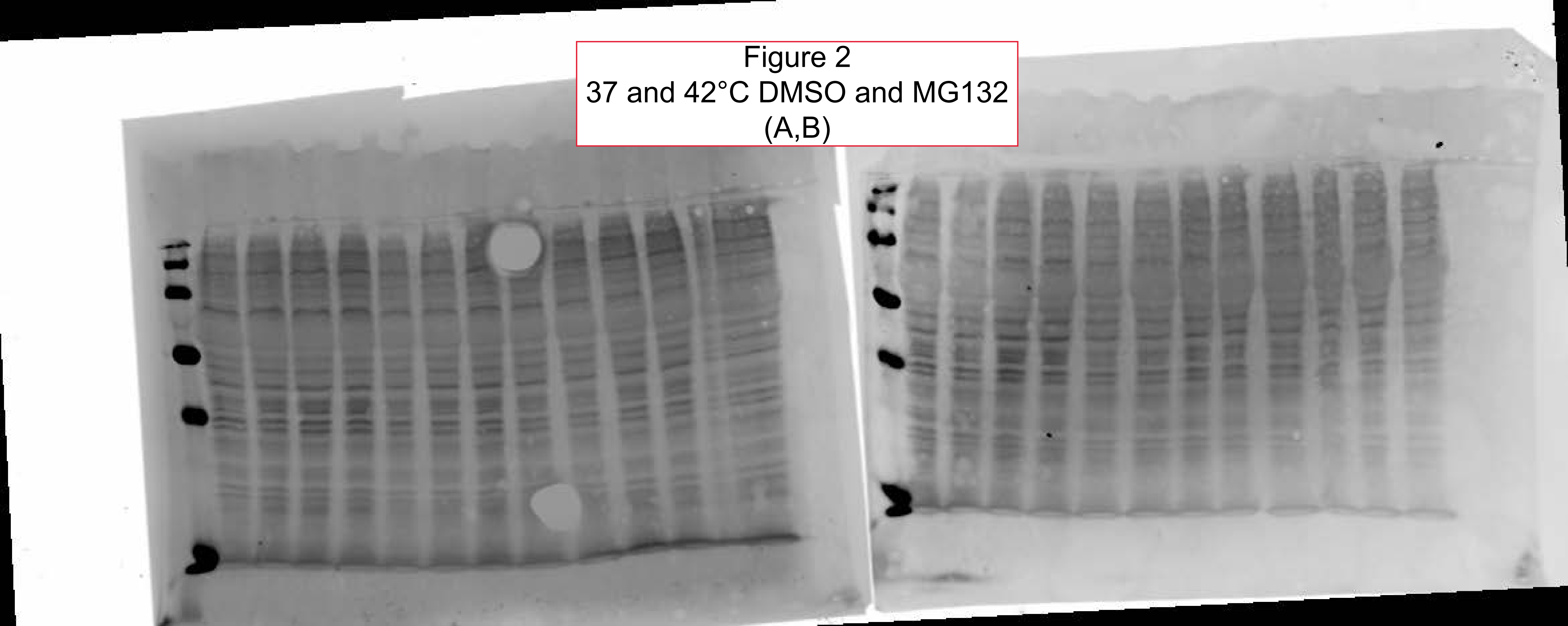

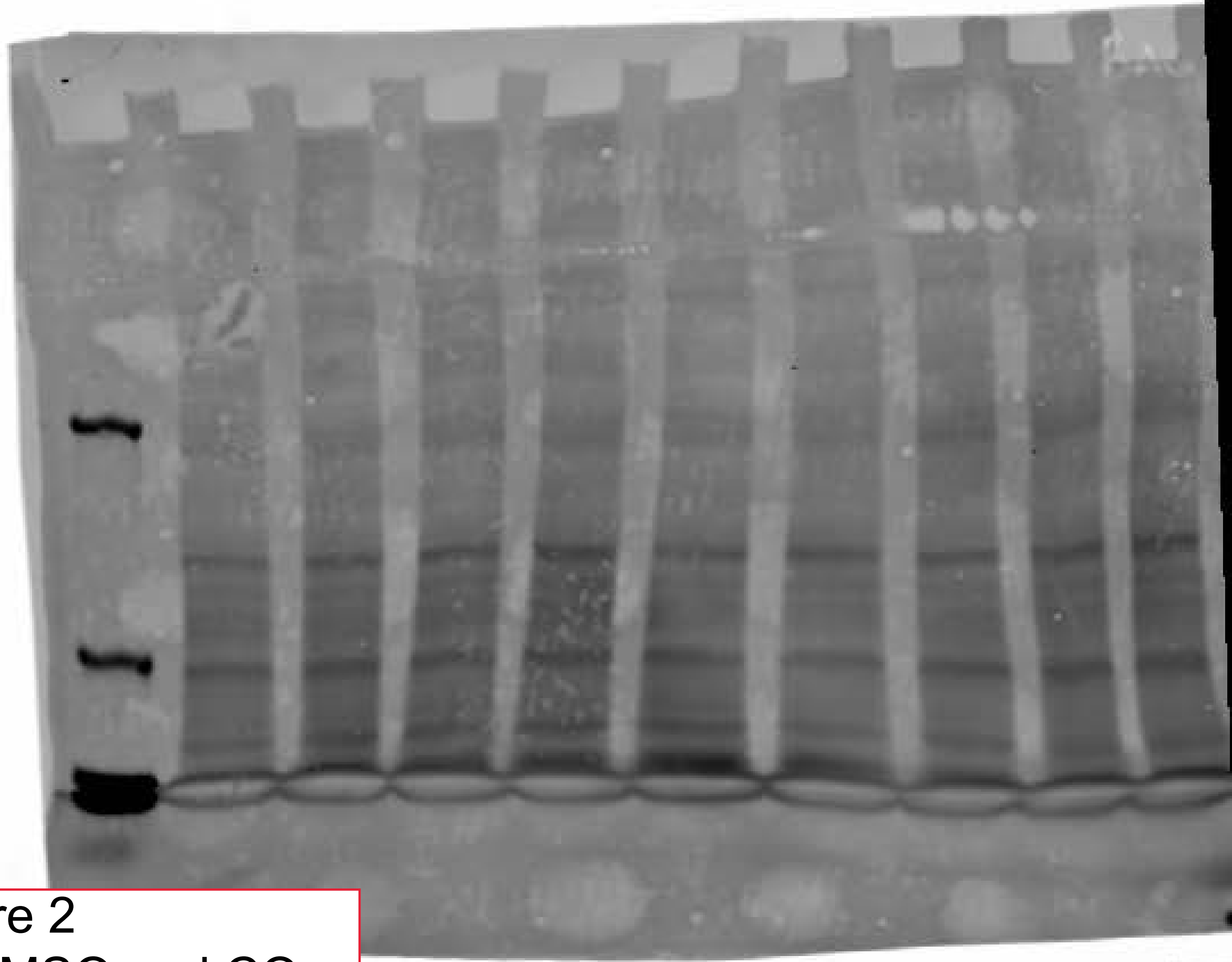

Figure 2
37 and 42°C DMSO and CQ
(C,D)

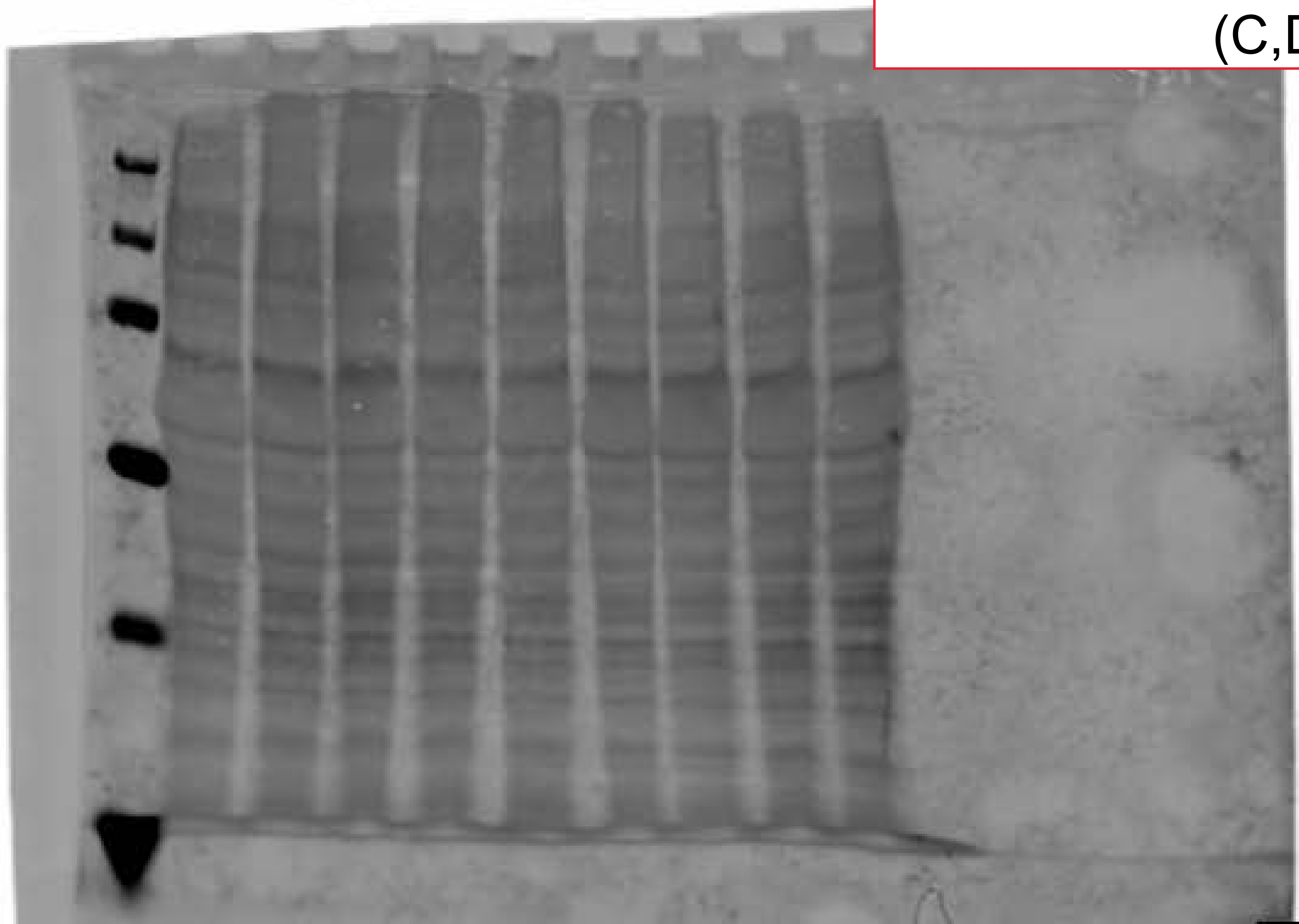

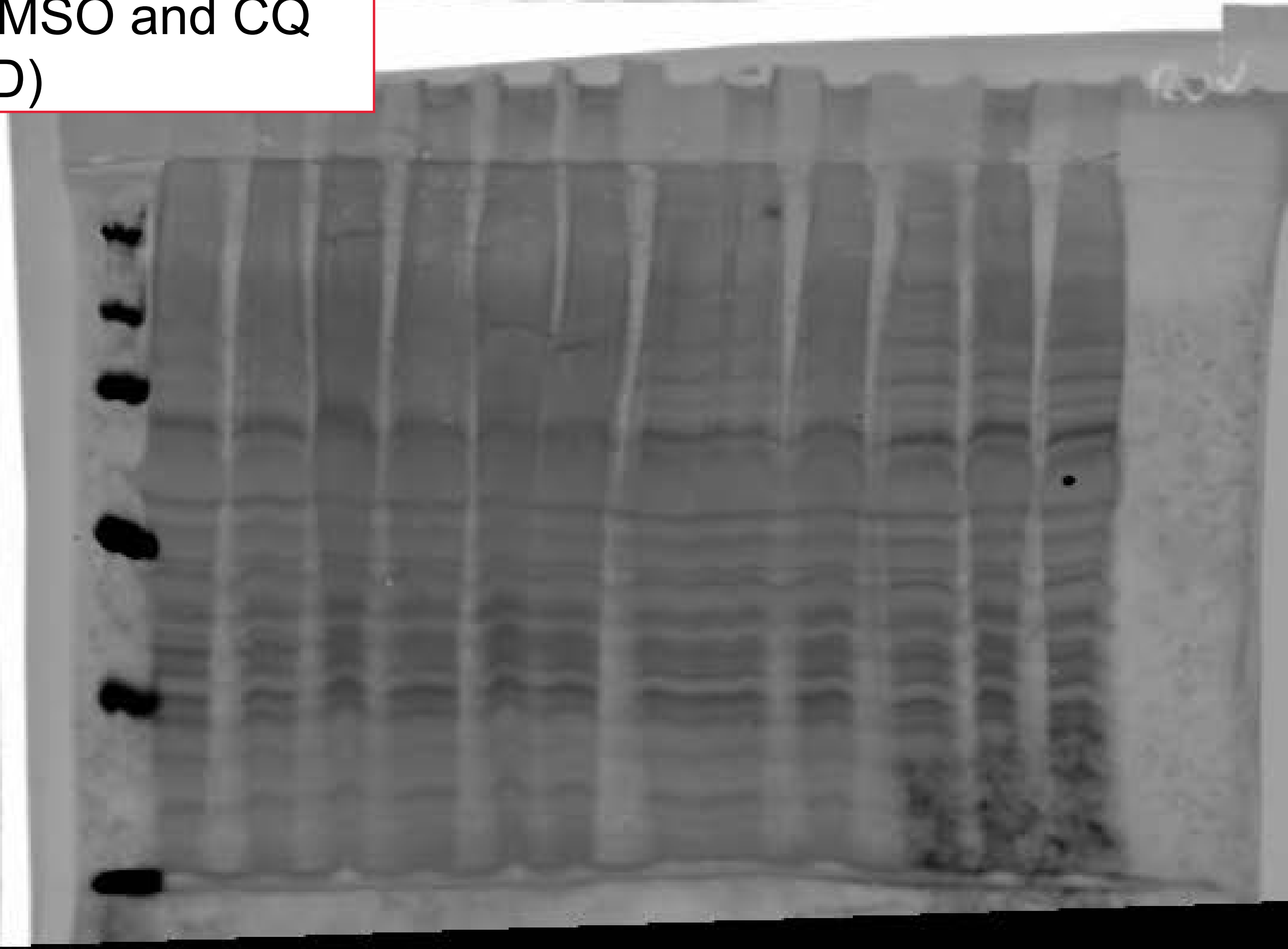

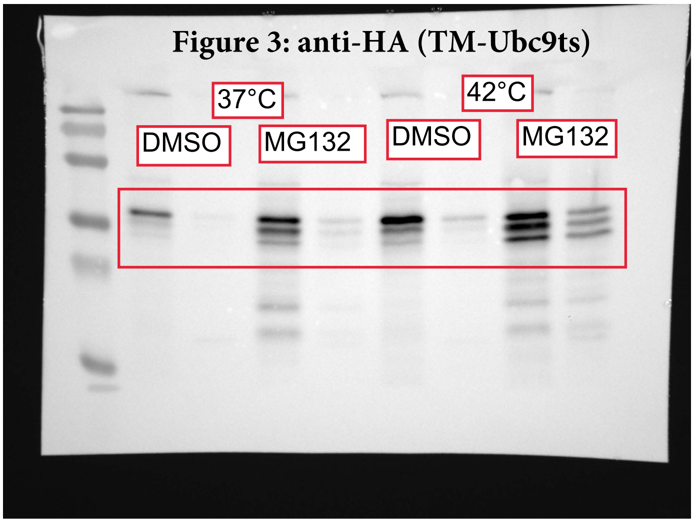

Figure 3: anti-HA (TM-Ubc9ts)

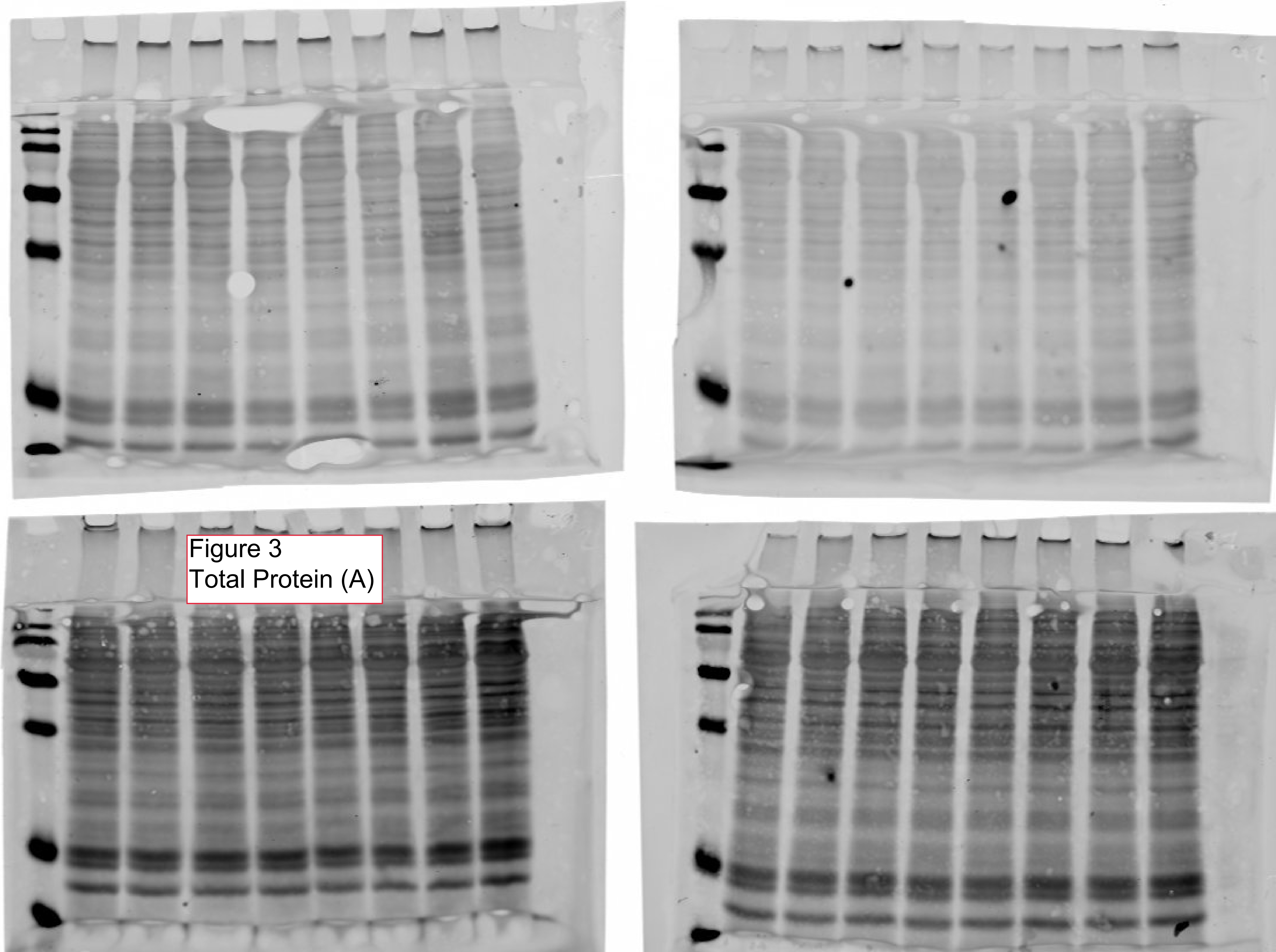

Figure 3
Total Protein (A)

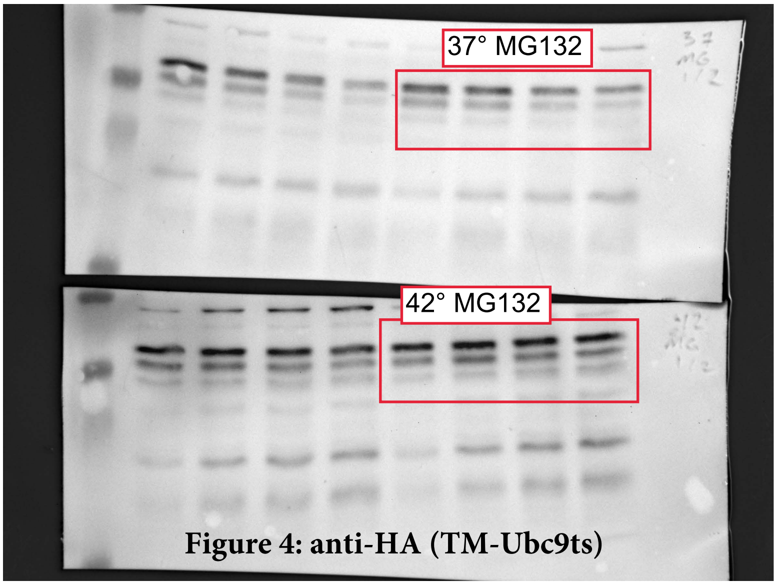

Figure 4: anti-HA (TM-Ubc9ts)

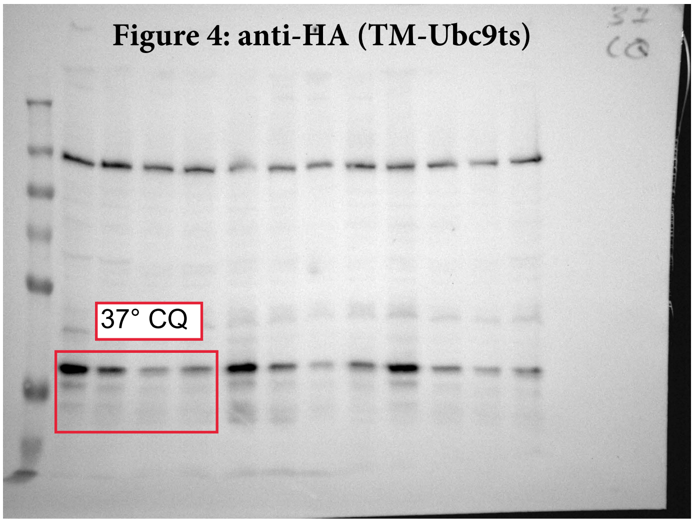

Figure 4: anti-HA (TM-Ubc9ts)

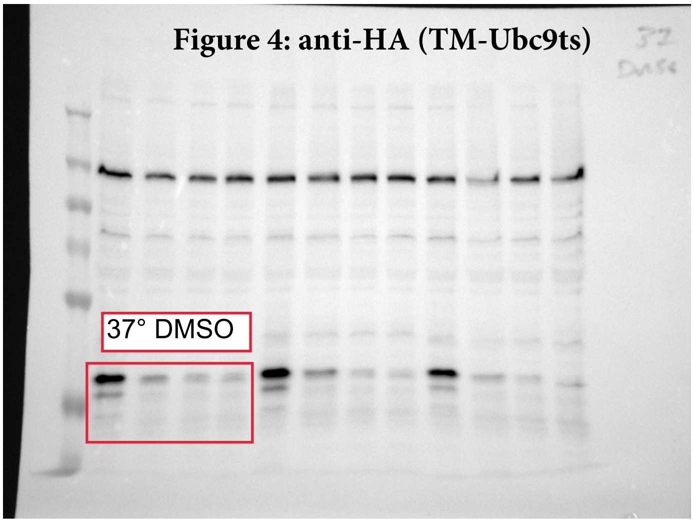

Figure 4: anti-HA (TM-Ubc9ts)

Figure 4: anti-HA (TM-Ubc9ts)

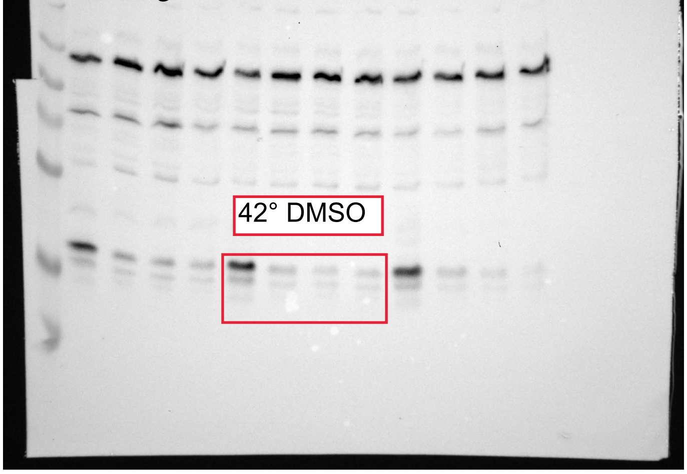

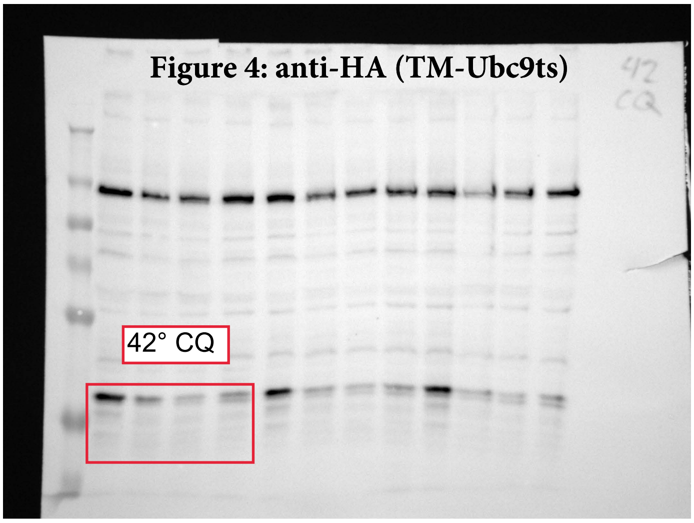

Figure 4: anti-HA (TM-Ubc9ts)

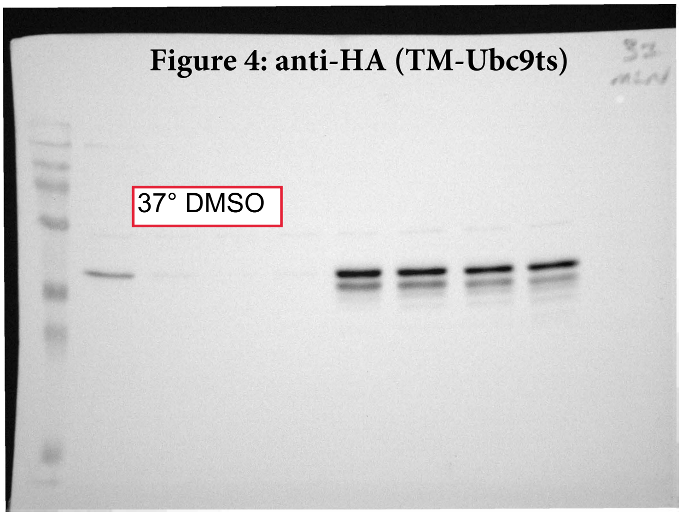

Figure 4: anti-HA (TM-Ubc9ts)

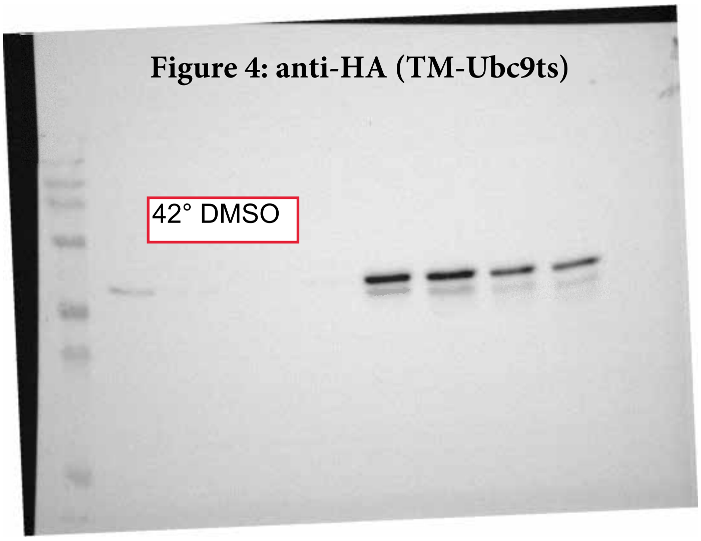

Figure 4: anti-HA (TM-Ubc9ts)

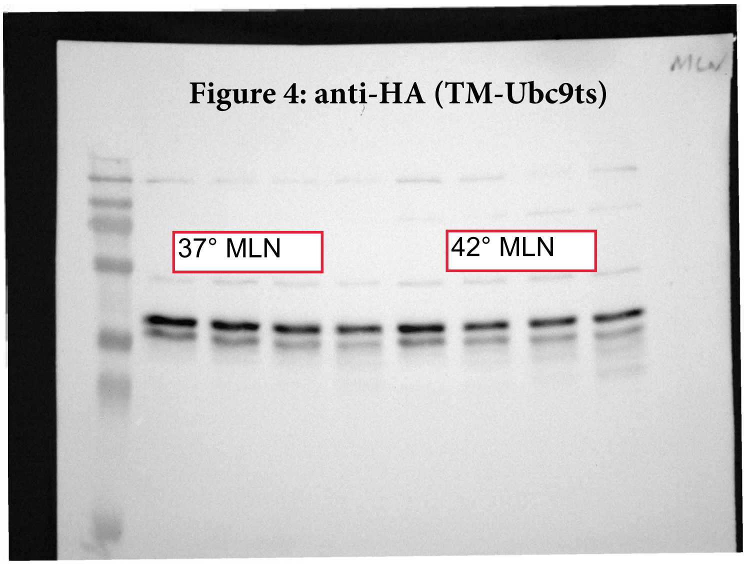
Figure 4: anti-HA (TM-Ubc9ts)
37° MLN
42° MLN
MLN

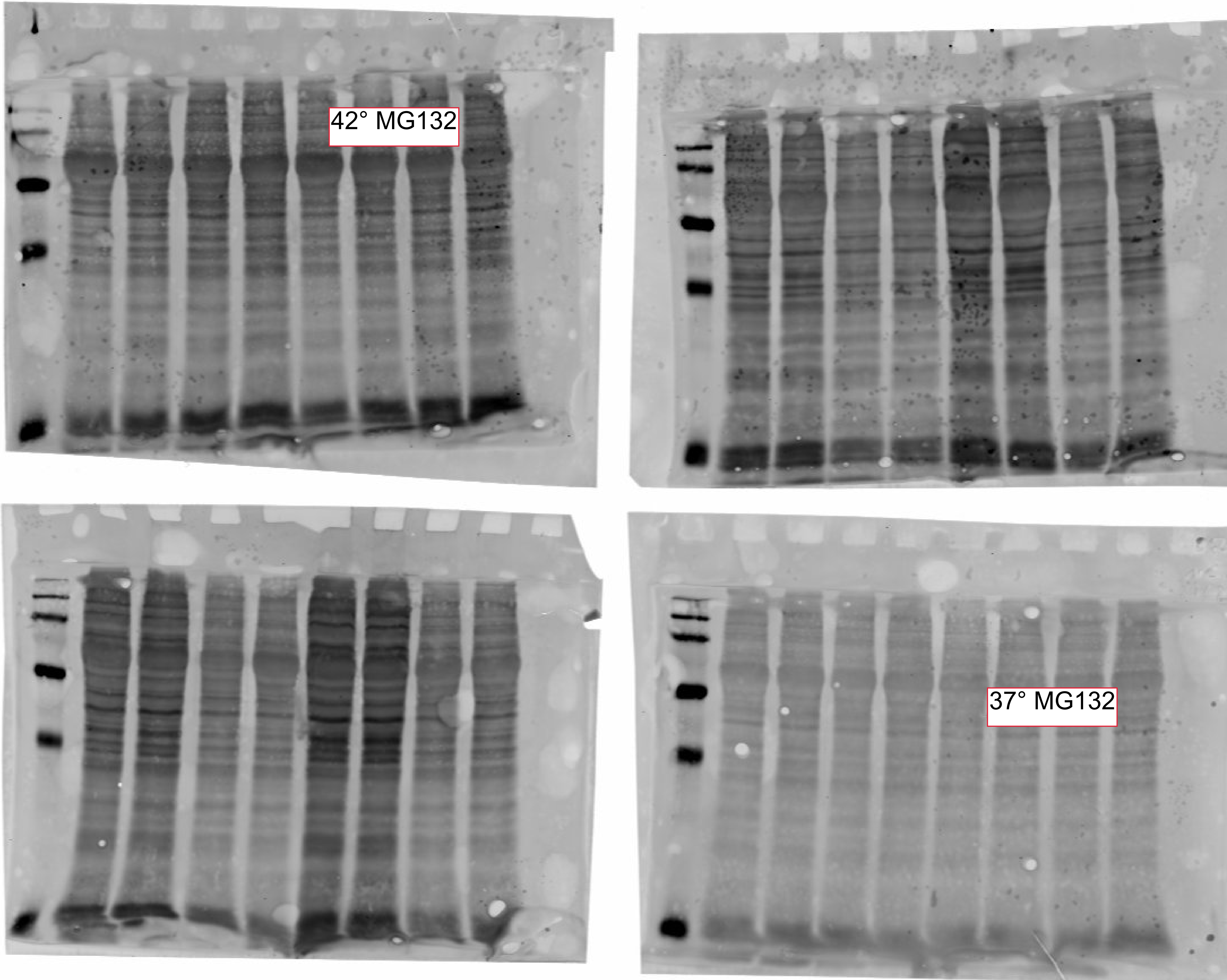
42° MG132
37° MG132

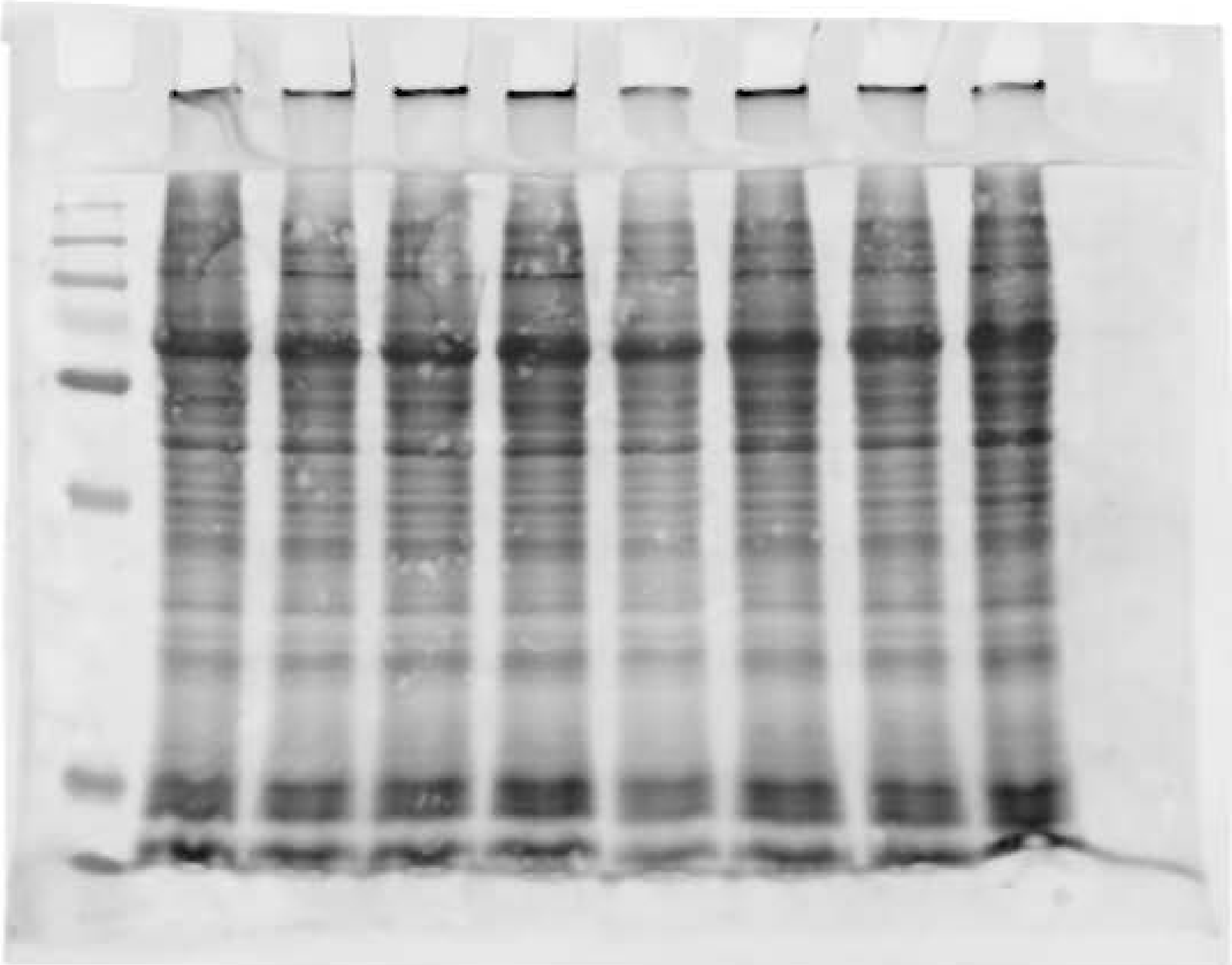

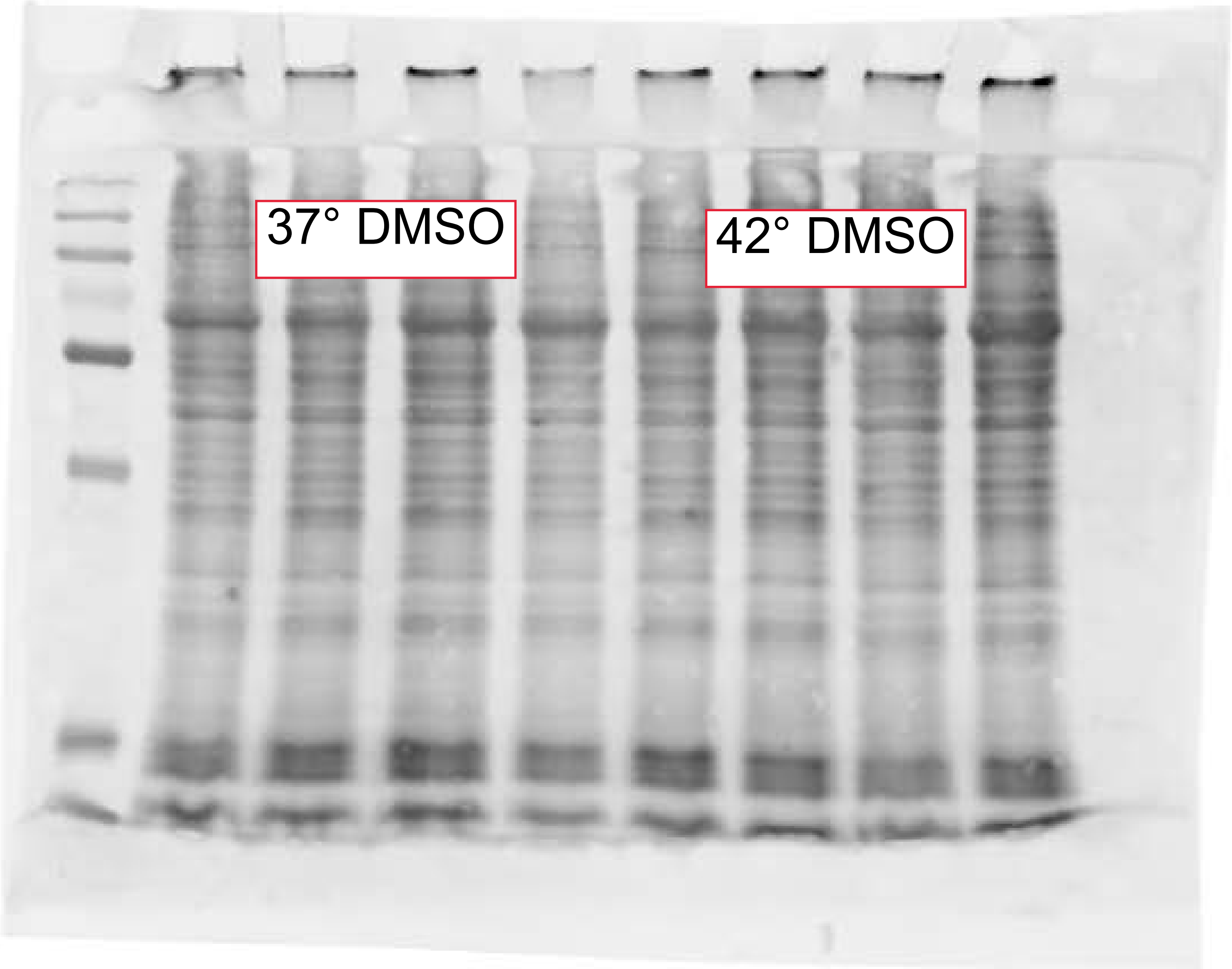
37° DMSO
42° DMSO

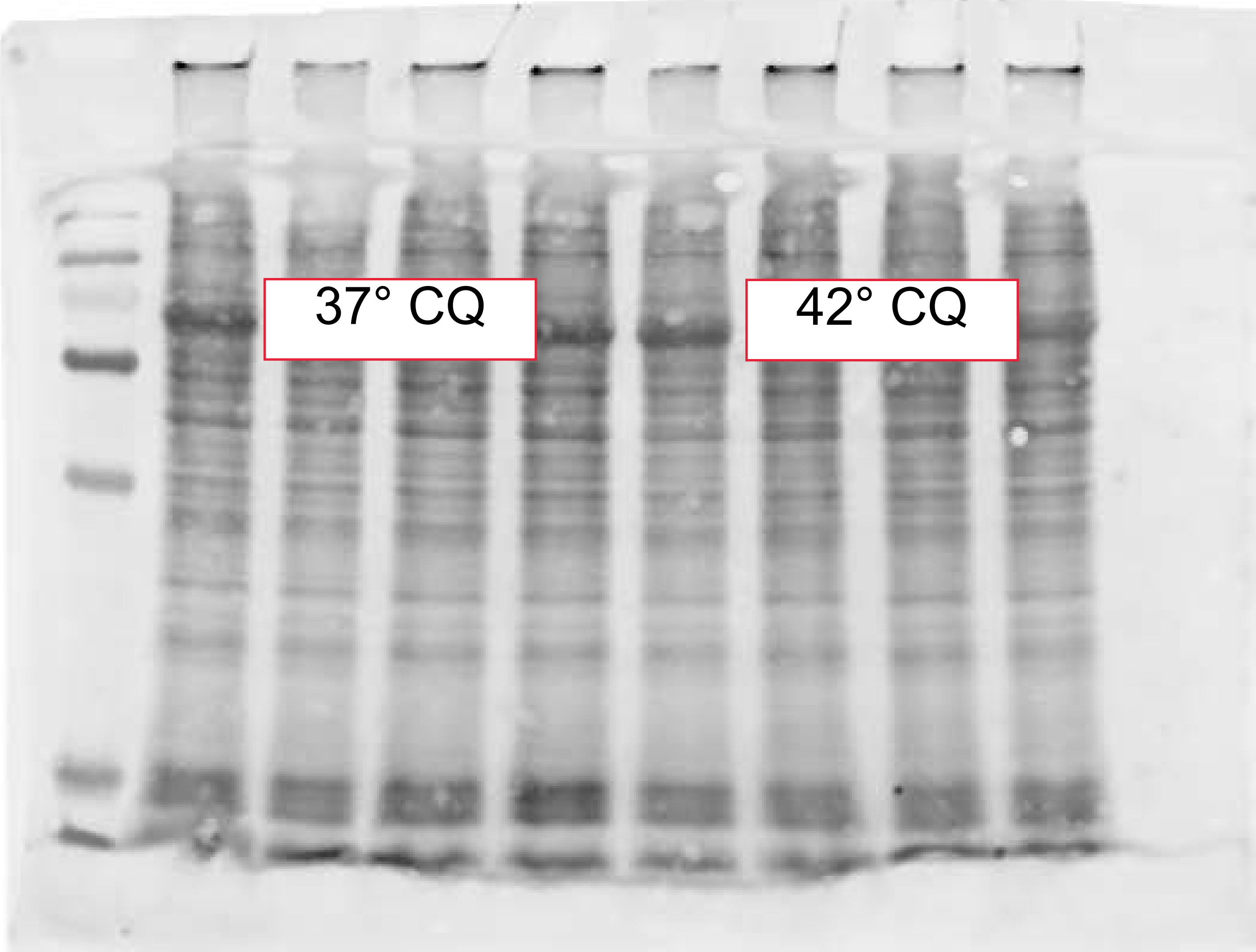
37° CQ
42° CQ

Figure 5: anti-HA (TM-Ubc9ts)

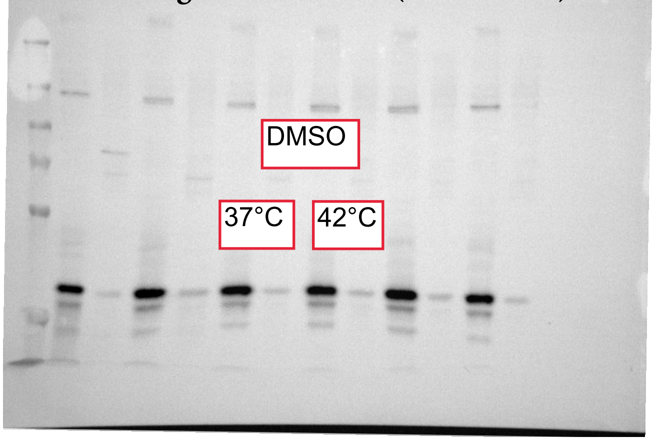

Figure 5: anti-HA (TM-Ubc9ts)

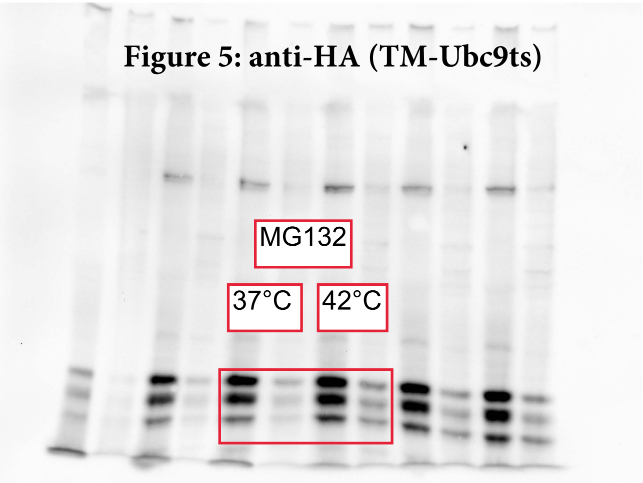

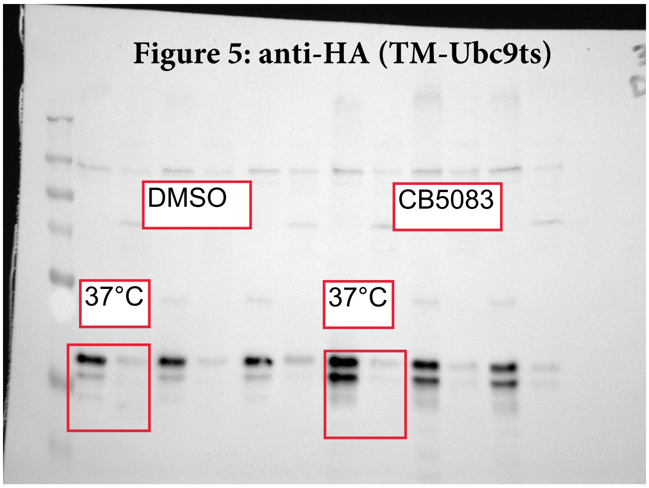

Figure 5: anti-HA (TM-Ubc9ts)

Figure 5: anti-HA (TM-Ubc9ts)

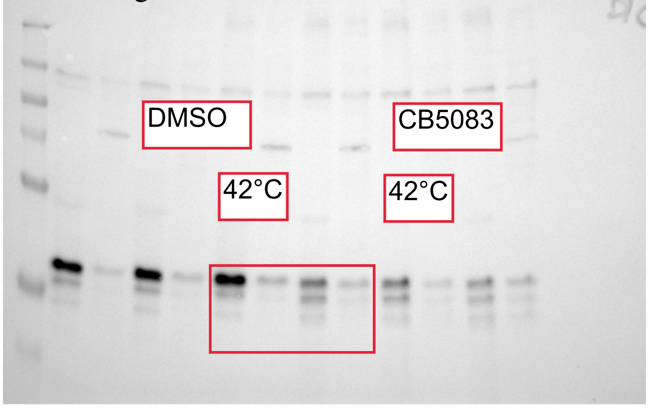

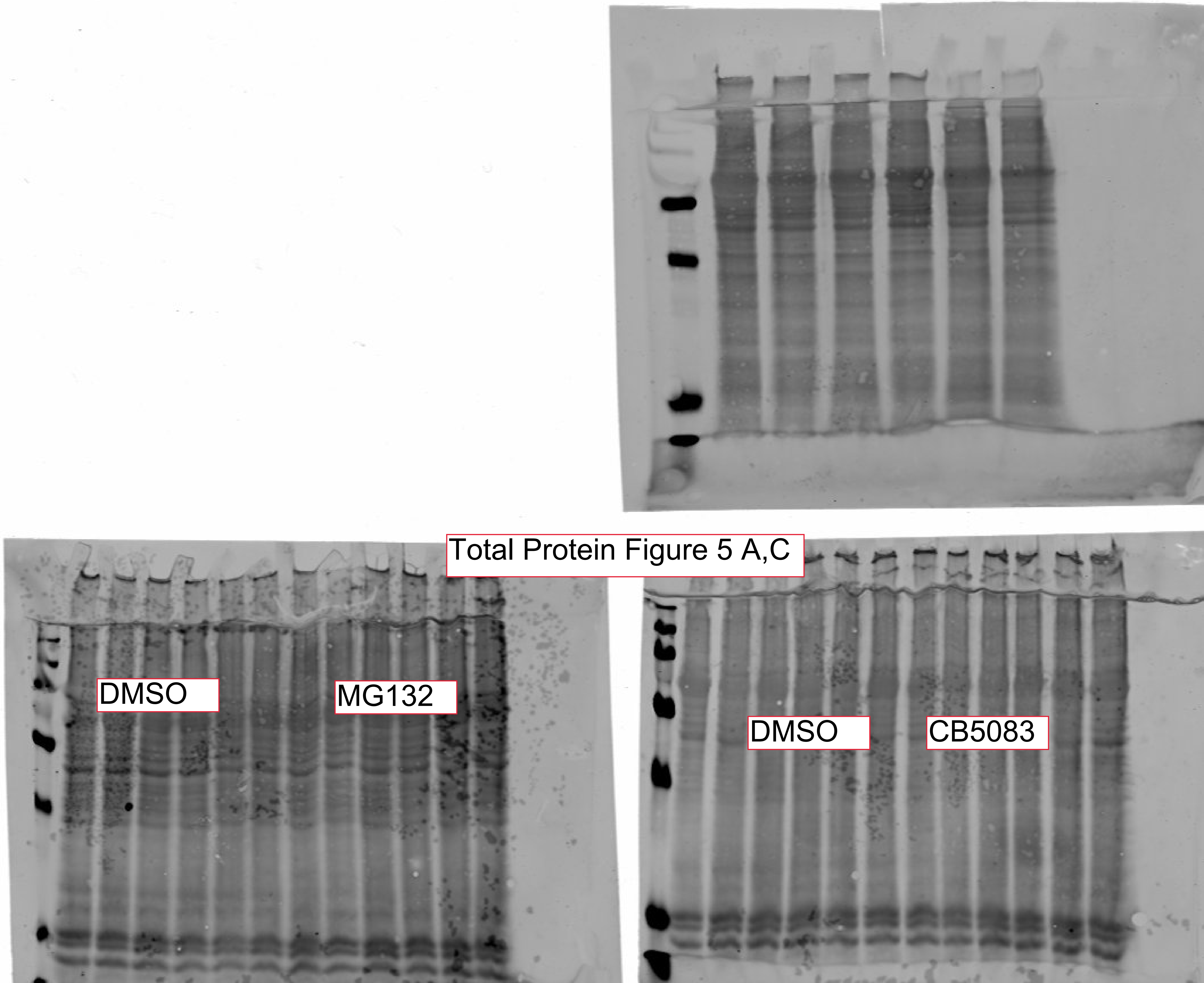
Total Protein Figure 5 A,C
DMSO
MG132
DMSO
CB5083

Figure 6: anti-HA (TM-Ubc9ts)

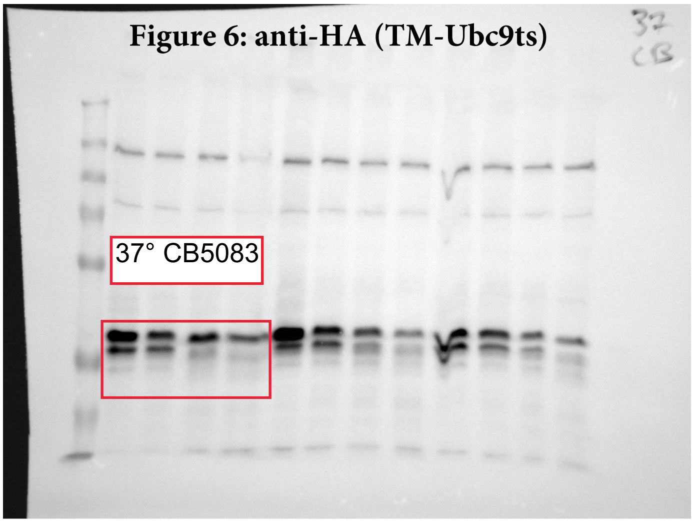

Figure 6: anti-HA (TM-Ubc9ts)

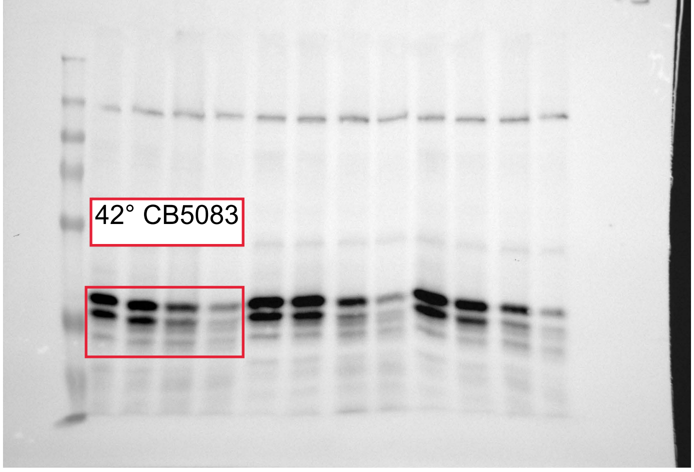

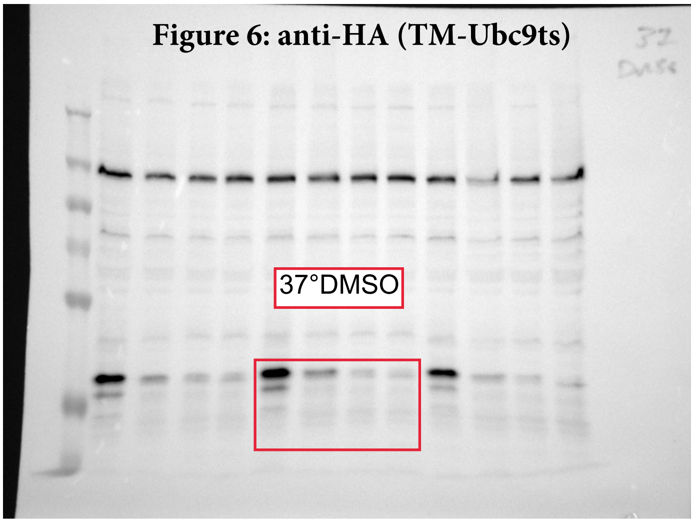

Figure 6: anti-HA (TM-Ubc9ts)

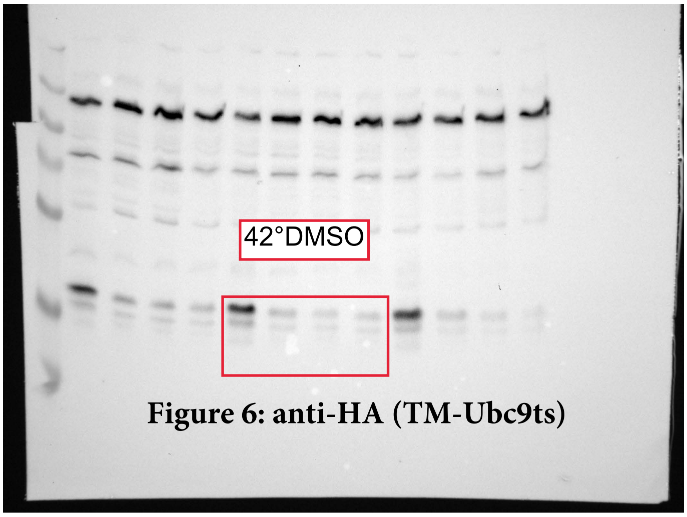

Figure 6: anti-HA (TM-Ubc9ts)

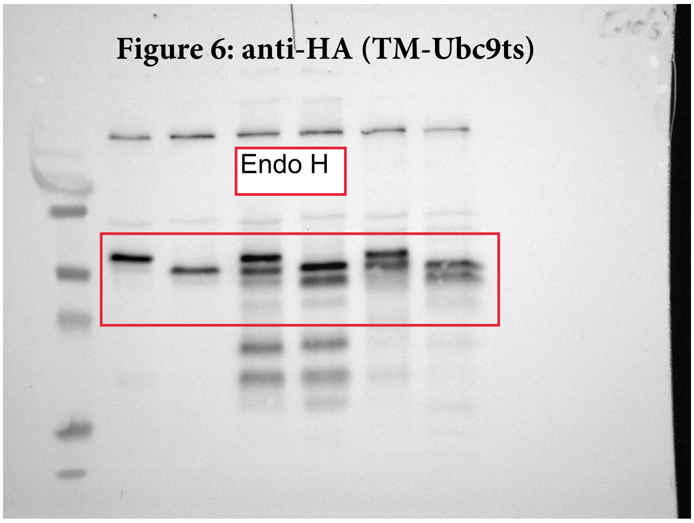
Figure 6: anti-HA (TM-Ubc9ts)
Endo H

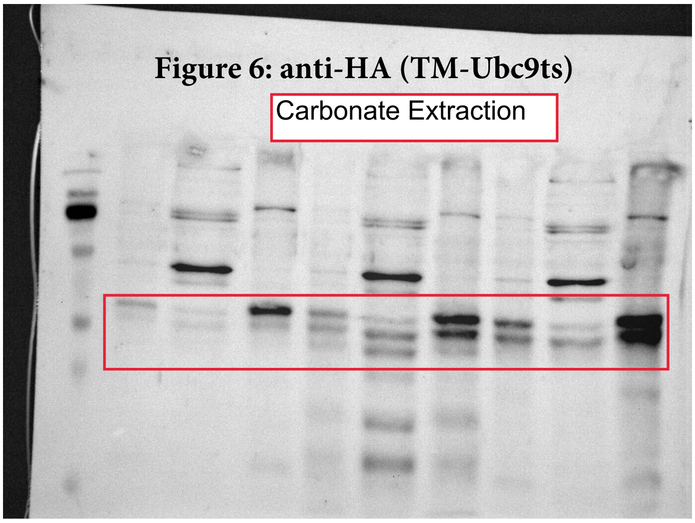

Figure 6: anti-HA (TM-Ubc9ts)

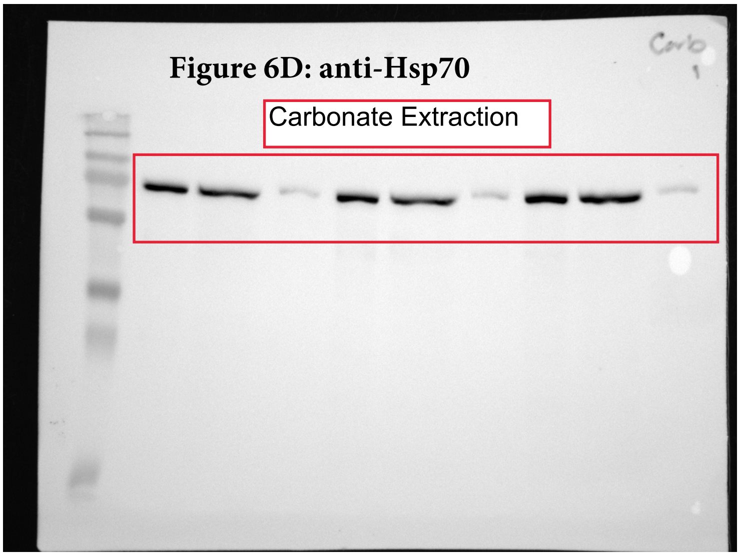

Figure 6D: anti-Hsp70

Total Protein
Figure 6 A,B

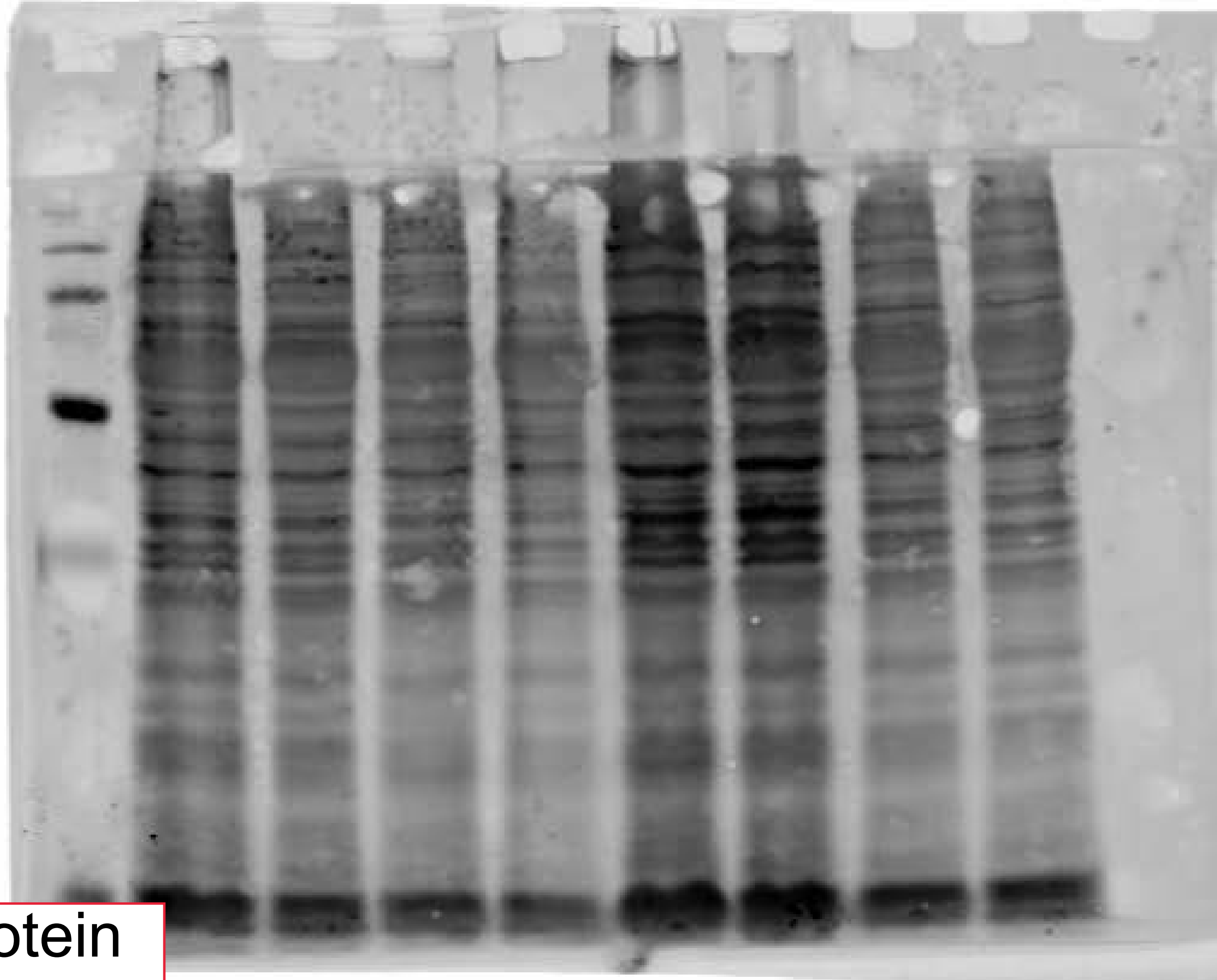

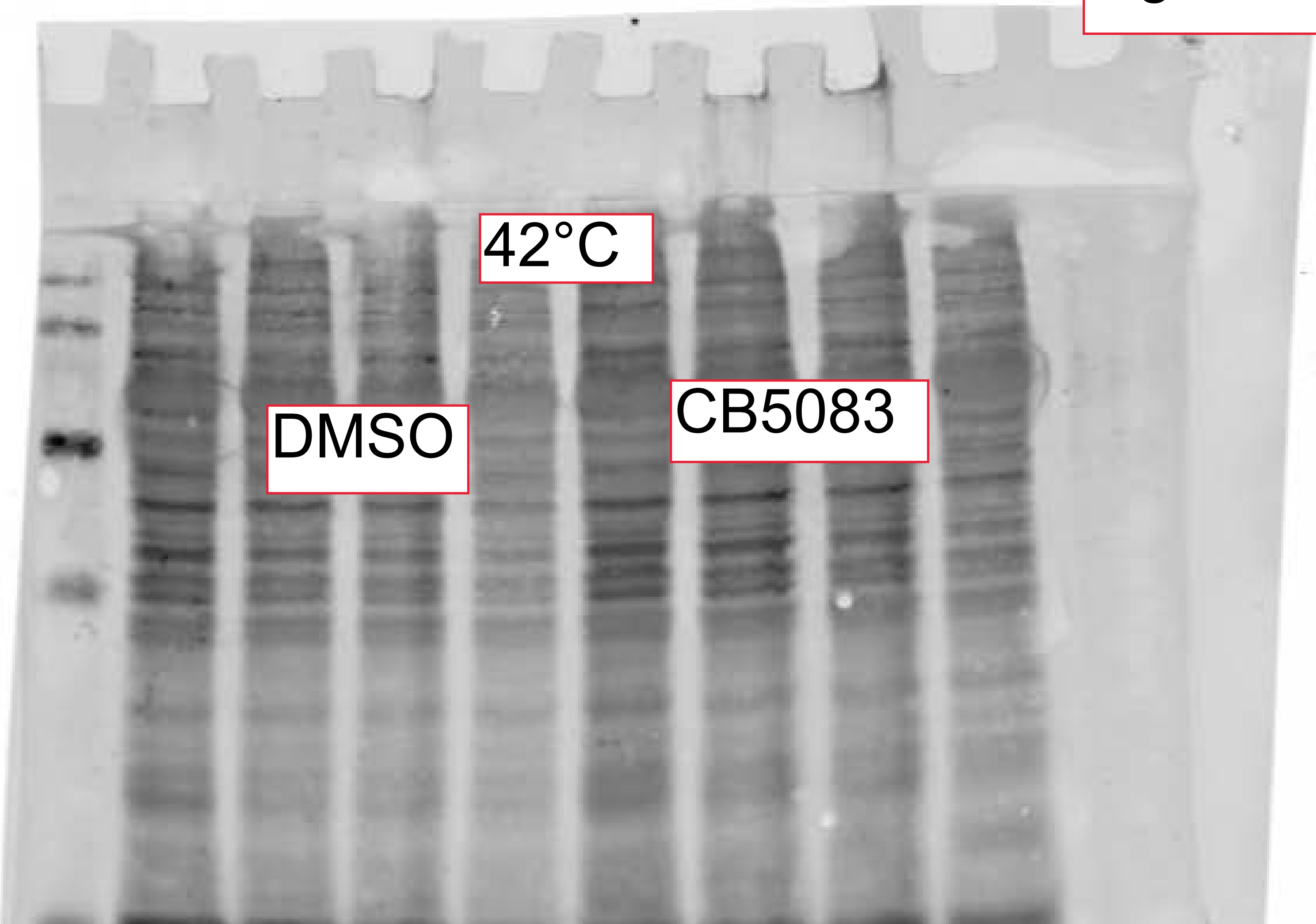

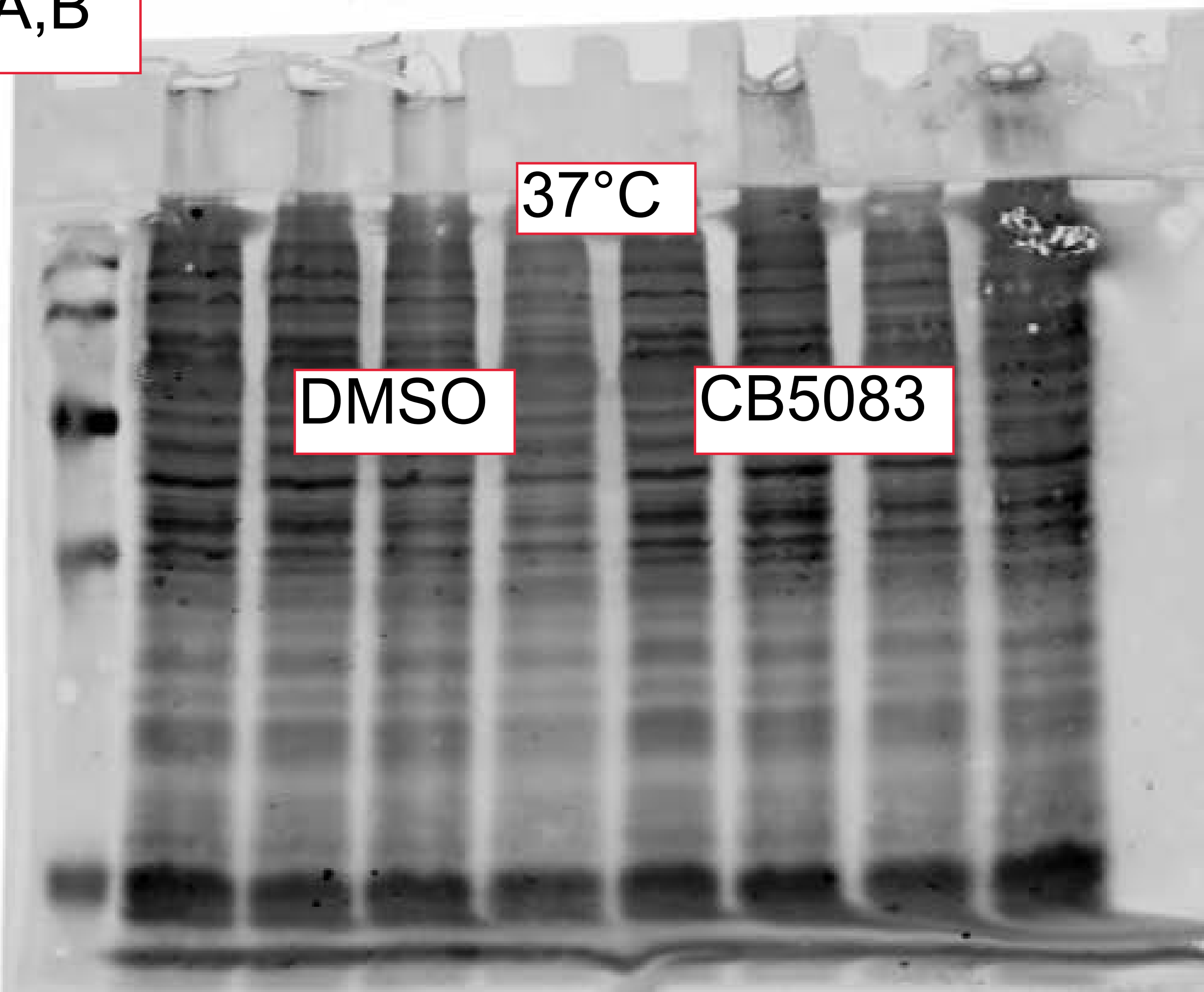

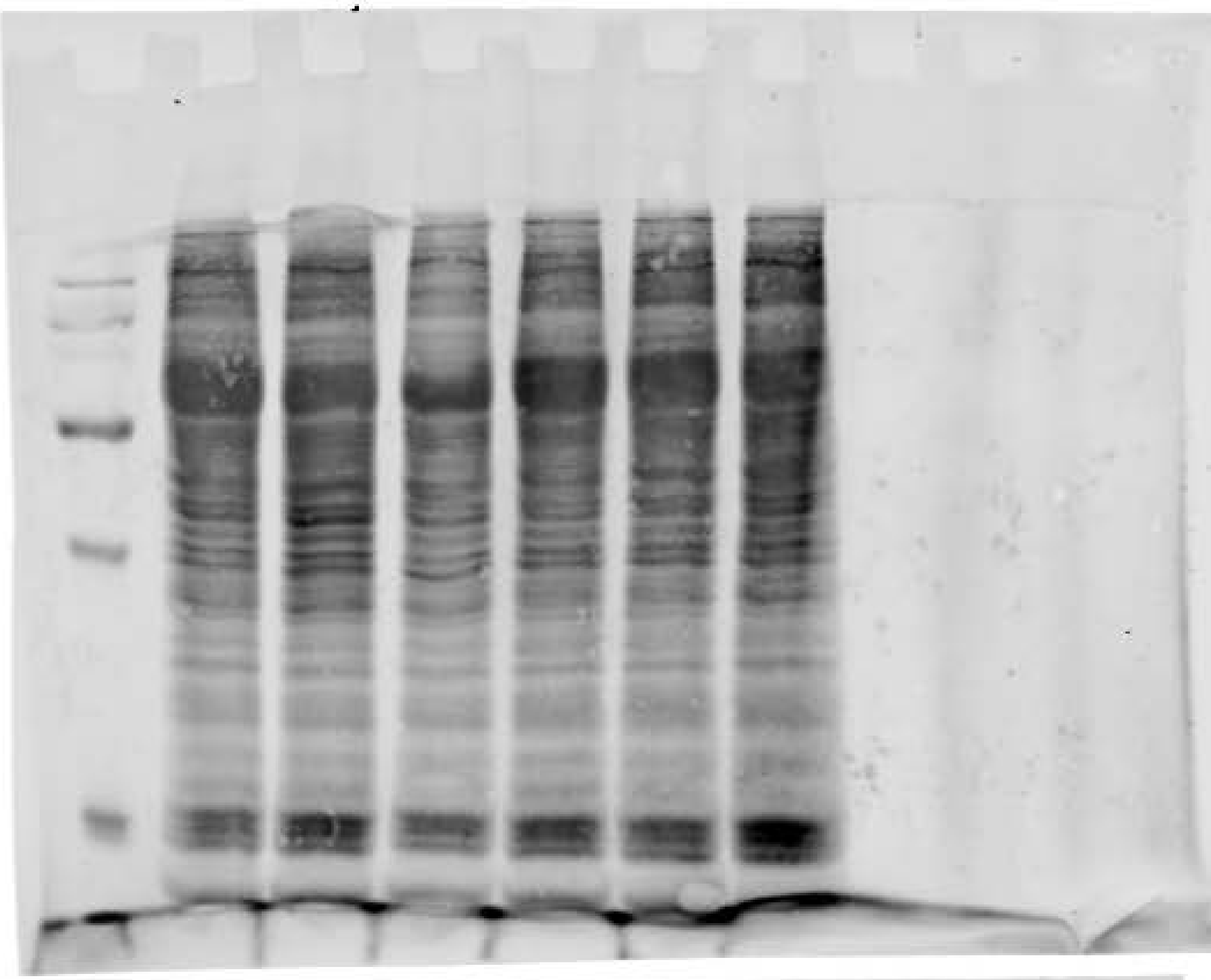

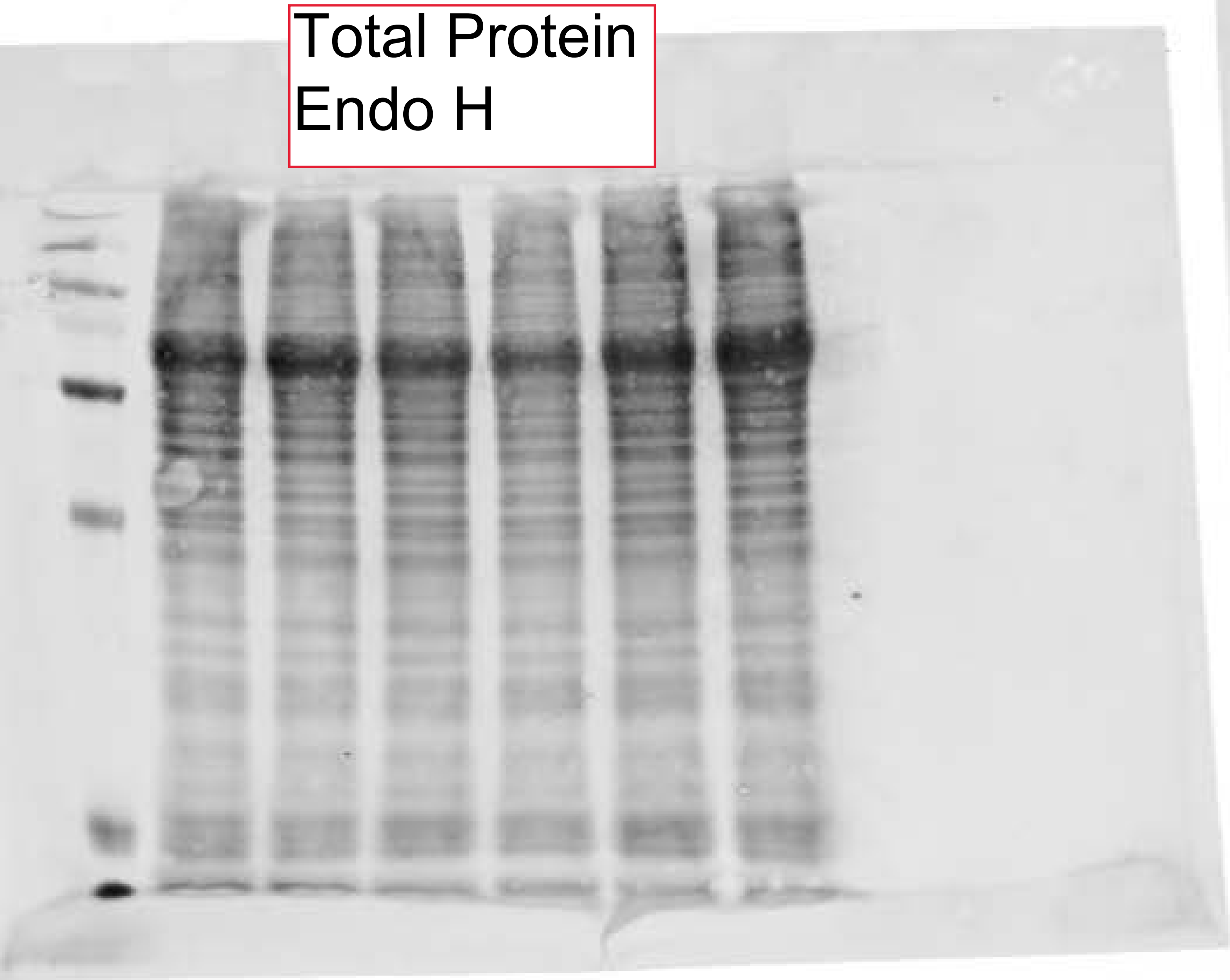
Total Protein
Endo H

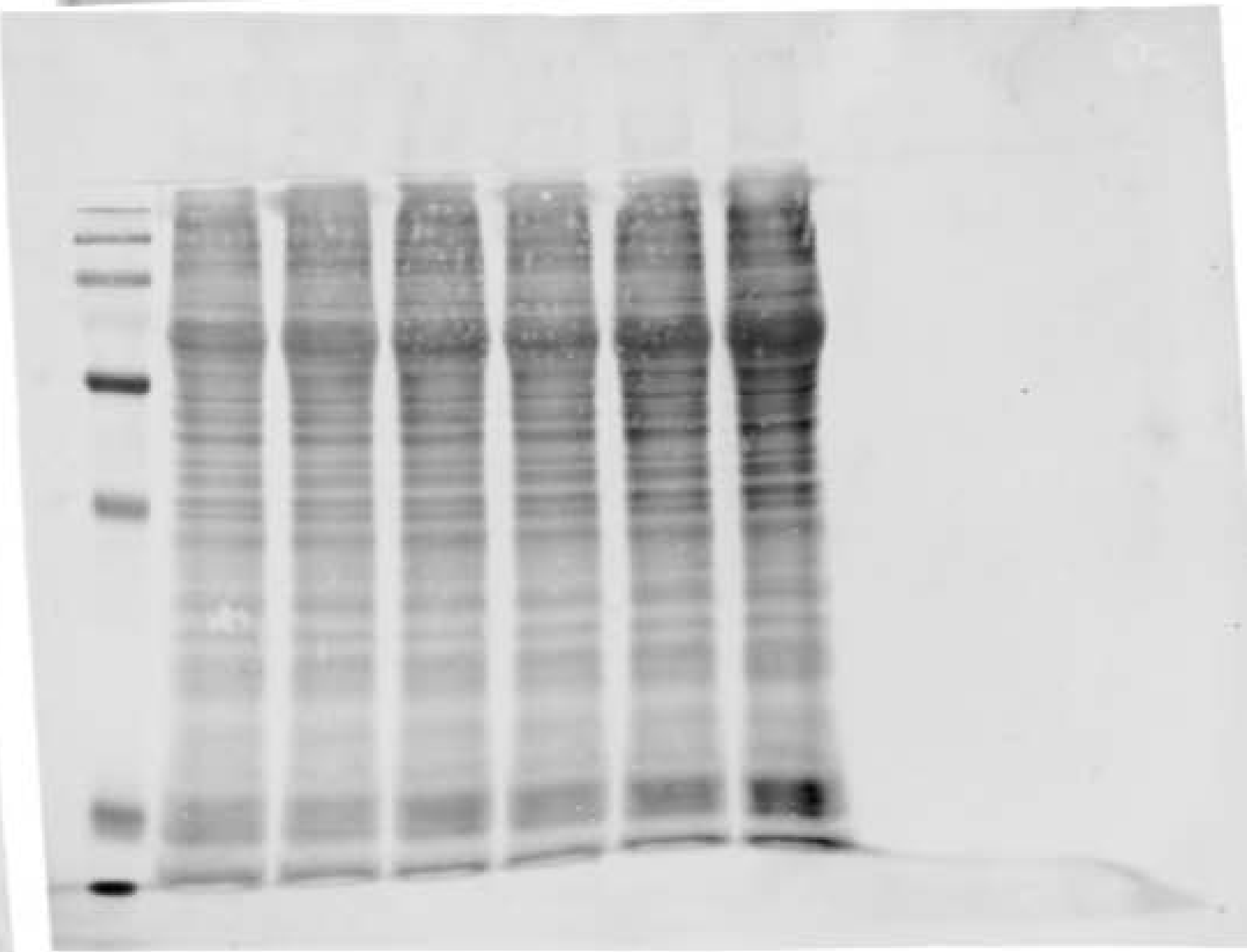

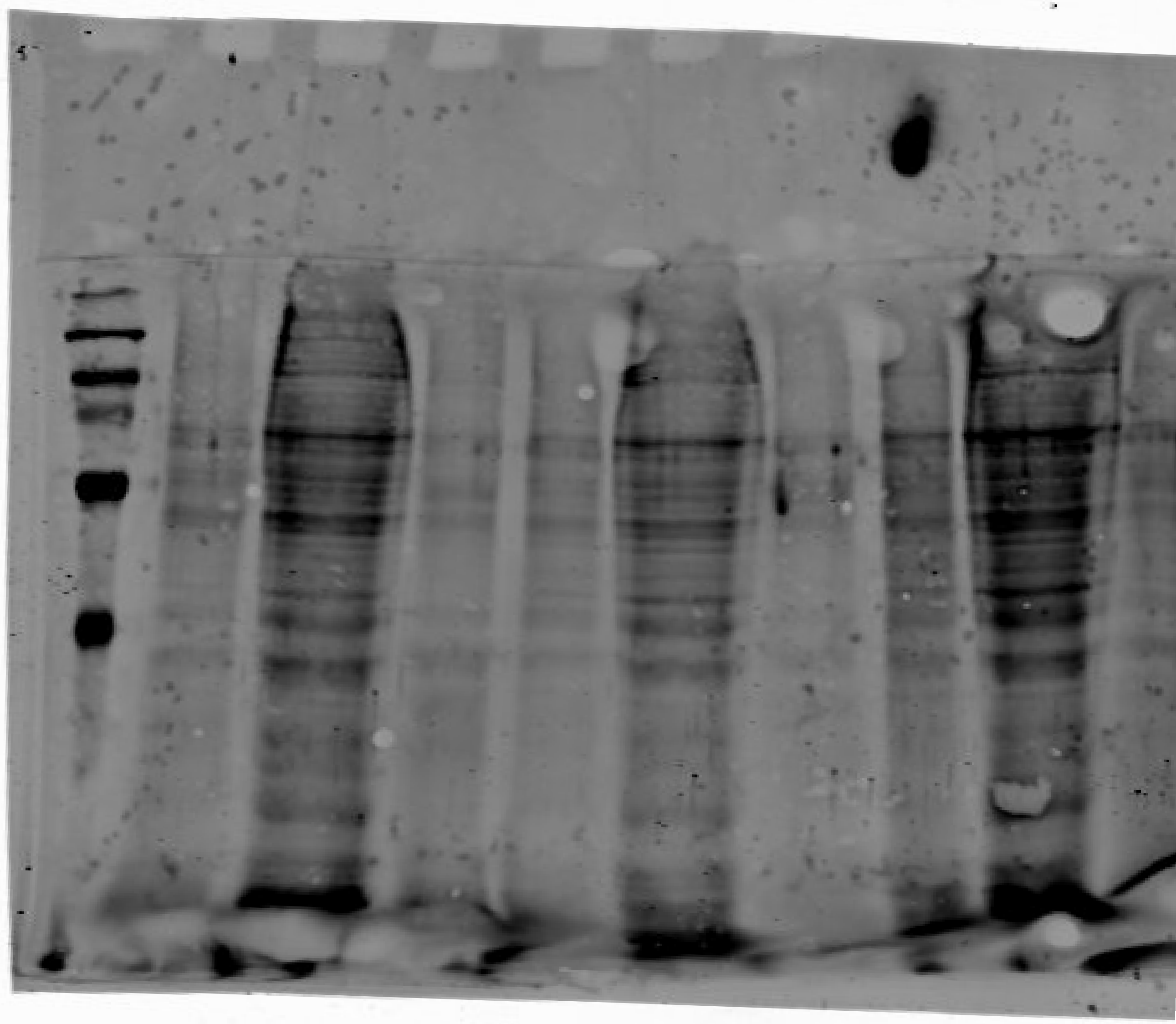

Total Protein
Carbonate Extraction: HA

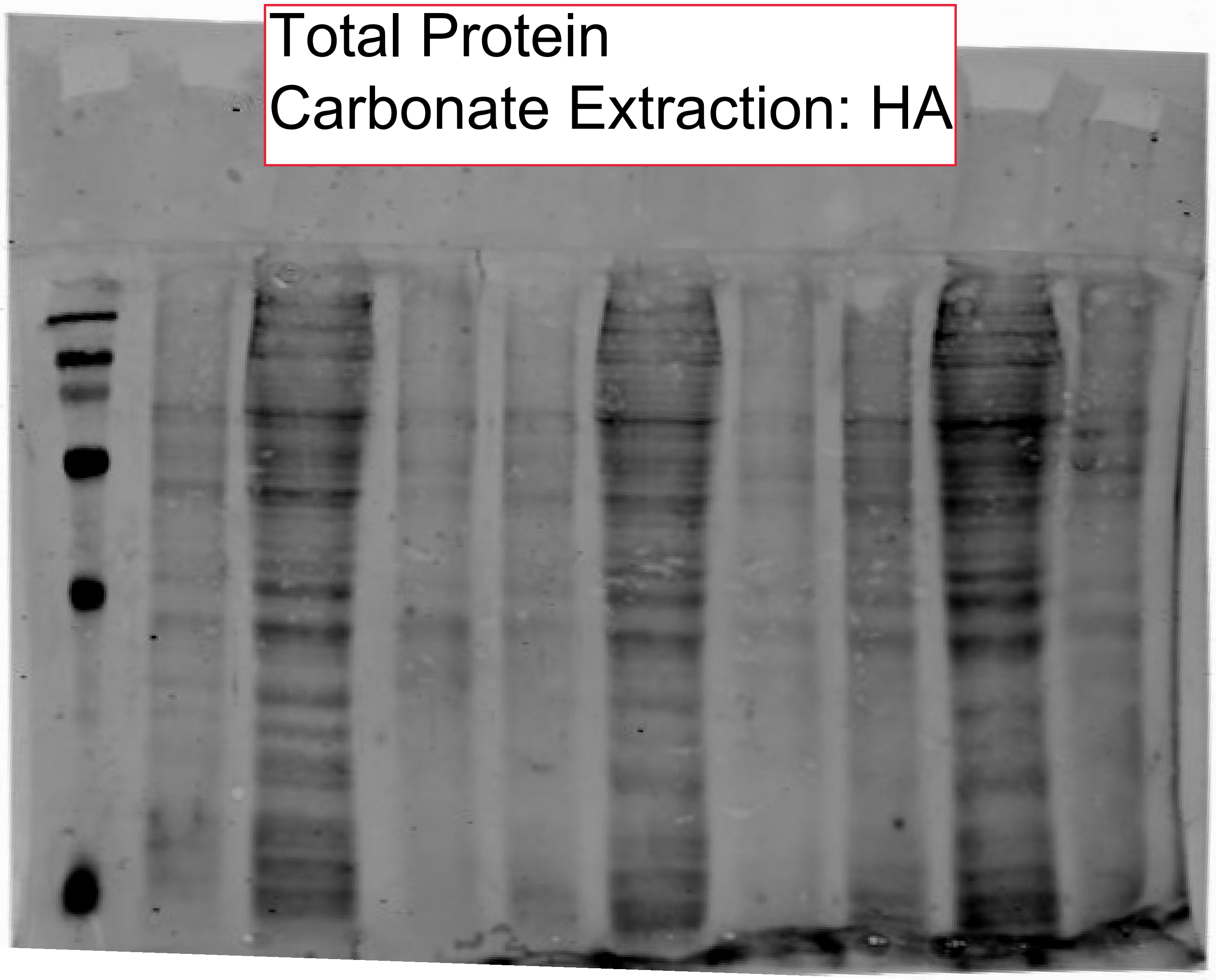

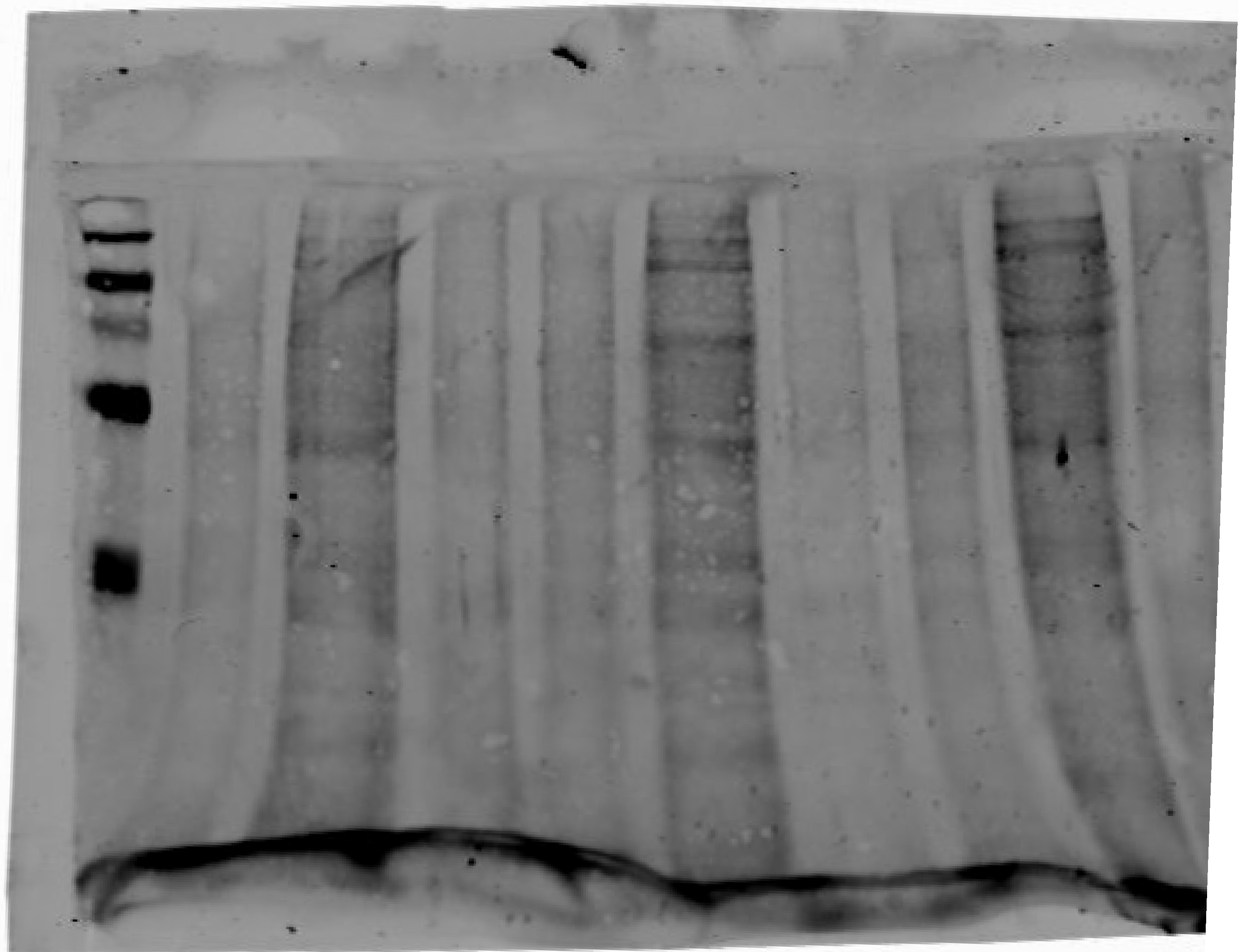

Total Protein: Figure 6D
Carbonate Extraction: Hsp70

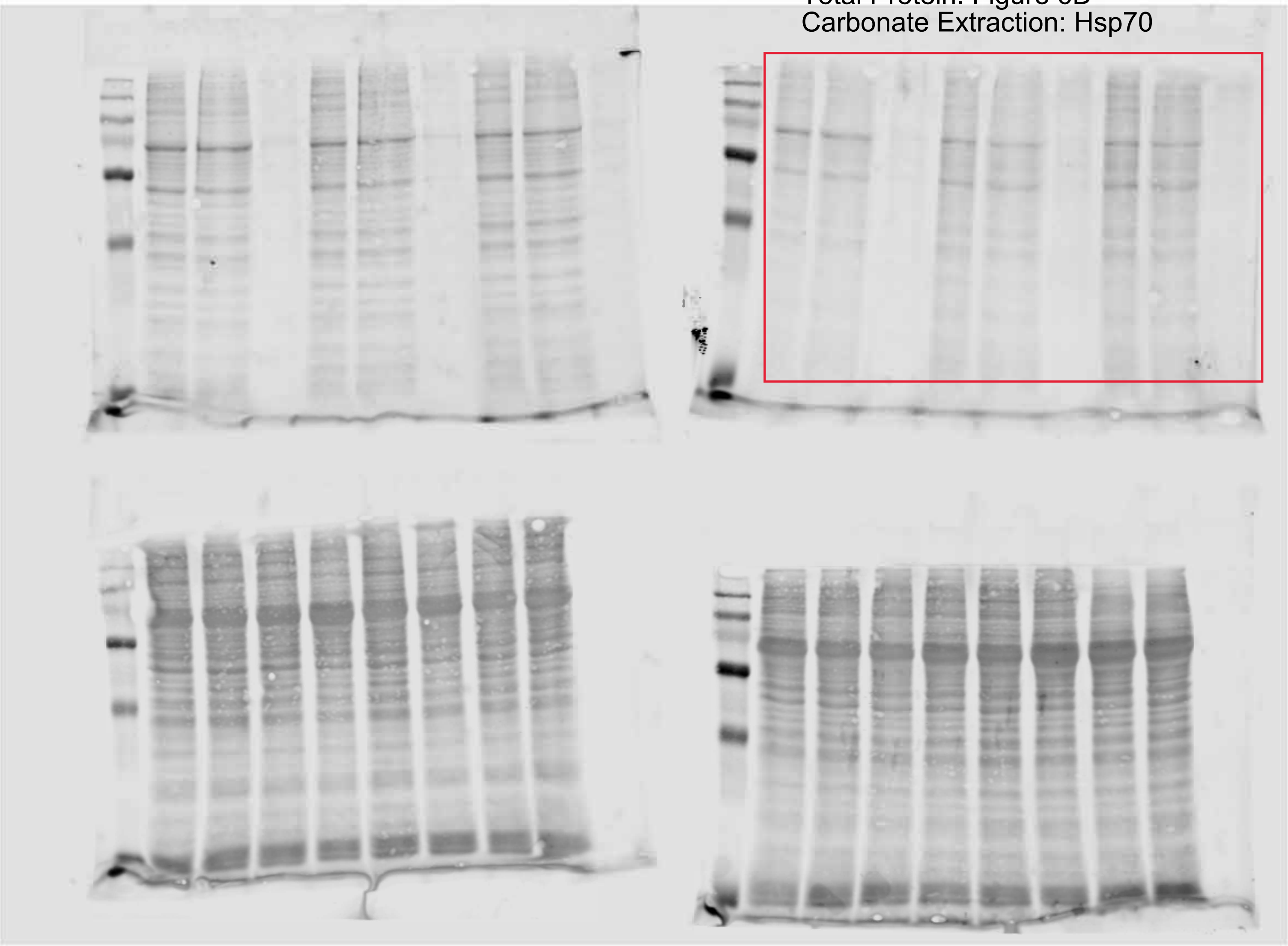

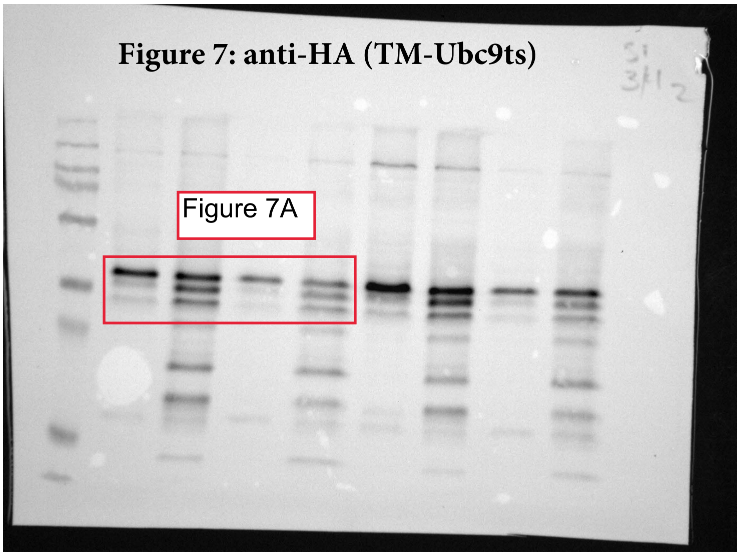

Figure 7: anti-HA (TM-Ubc9ts)
Figure 7A

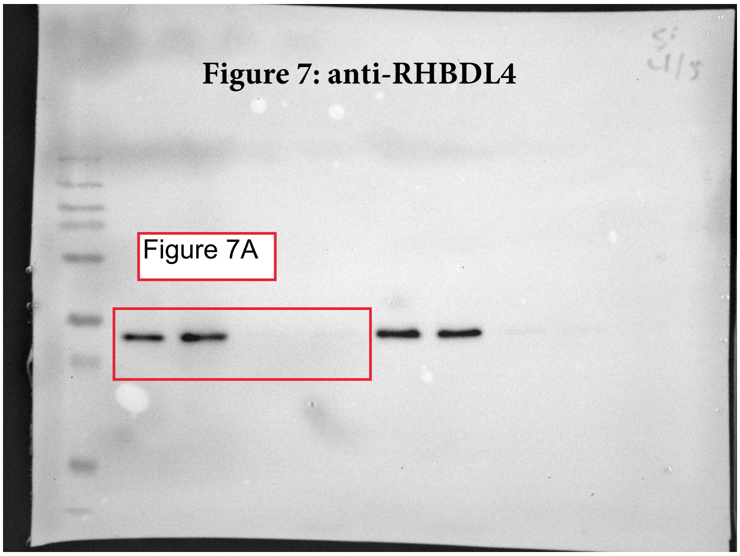
Figure 7: anti-RHBDL4
Figure 7A

Figure 7: Total Protein

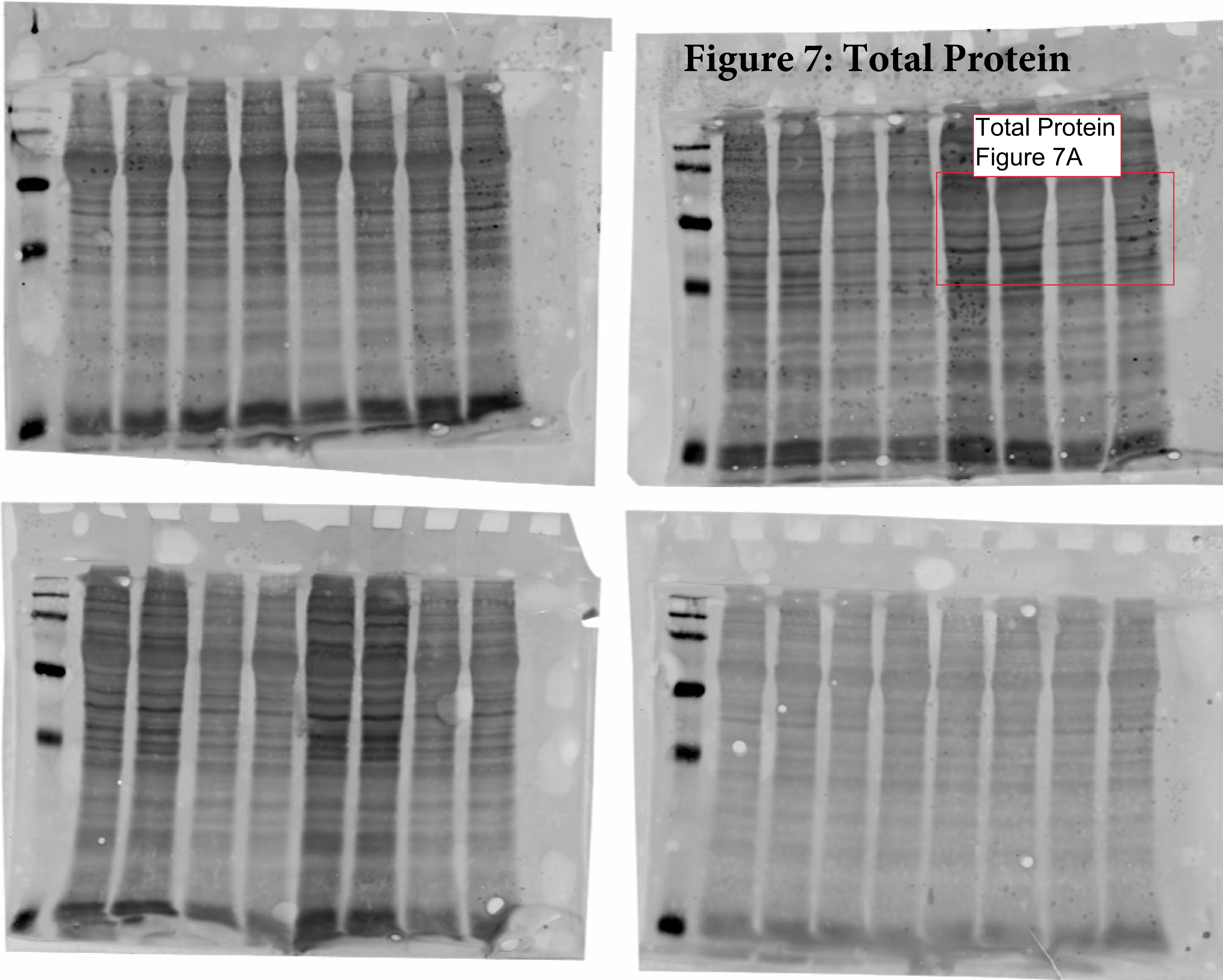

Figure 7: anti-FLAG (MHC202)

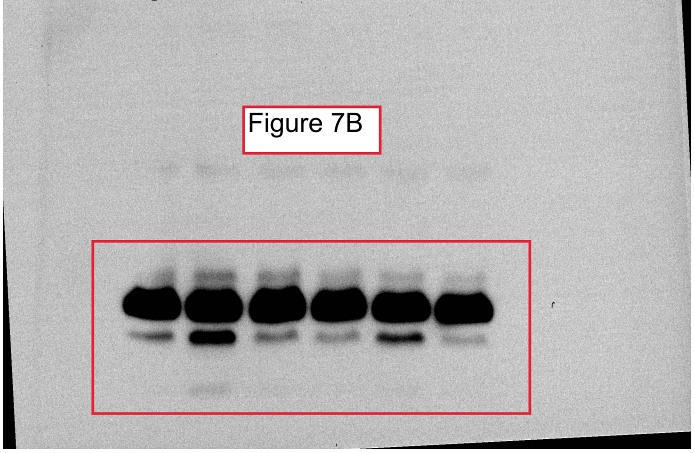

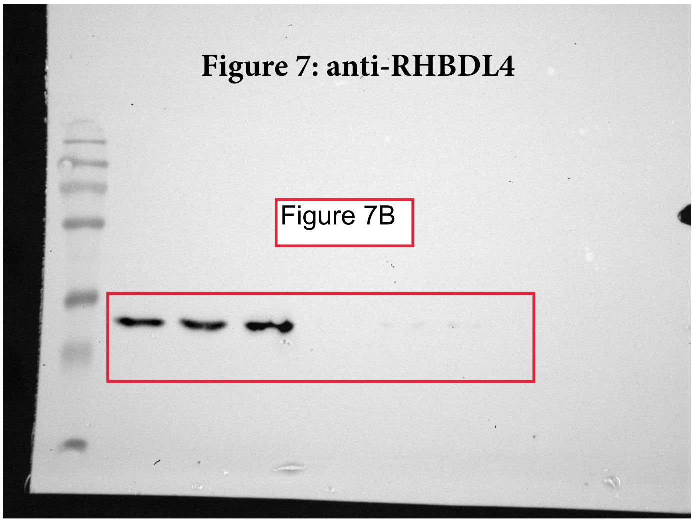

Figure 7: anti-RHBDL4

Figure 7: anti-GFP (Ubc9ts-GFP)

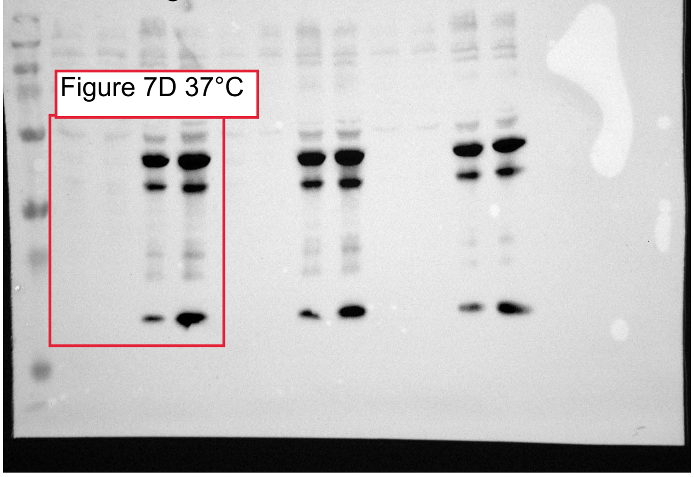

Figure 7: anti-GFP (Ubc9ts-GFP)

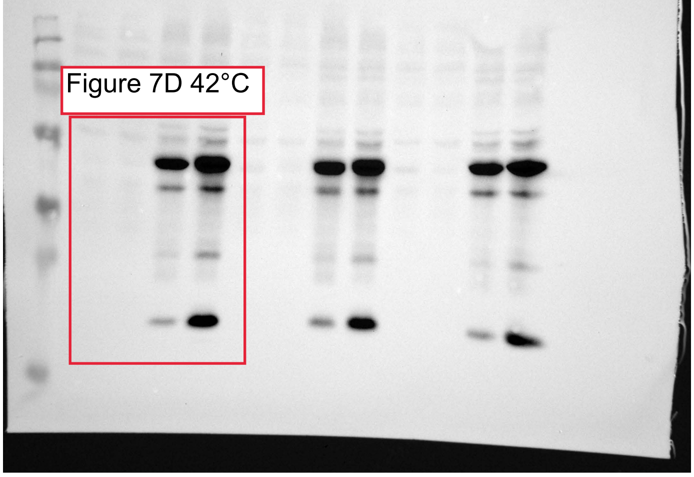

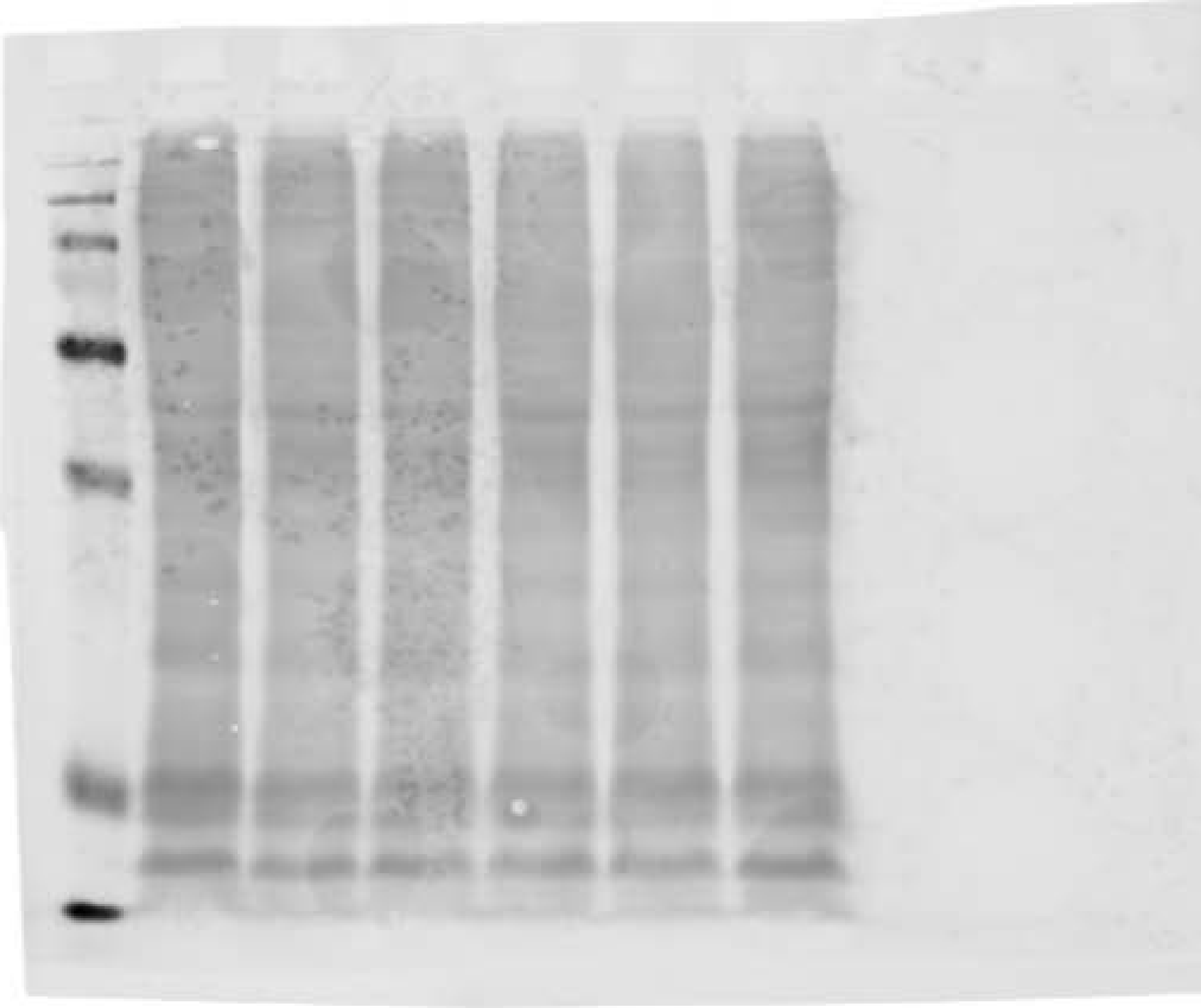

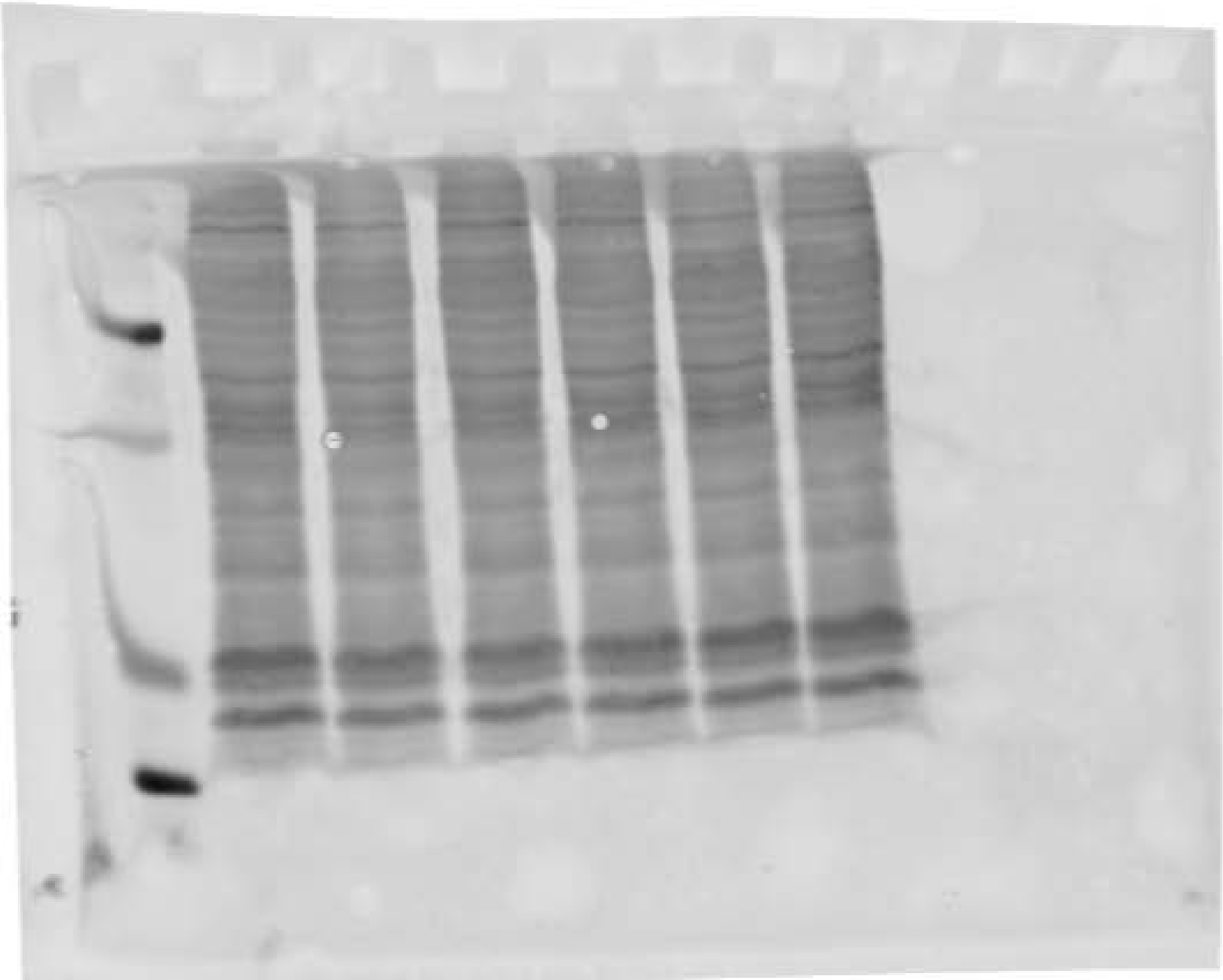

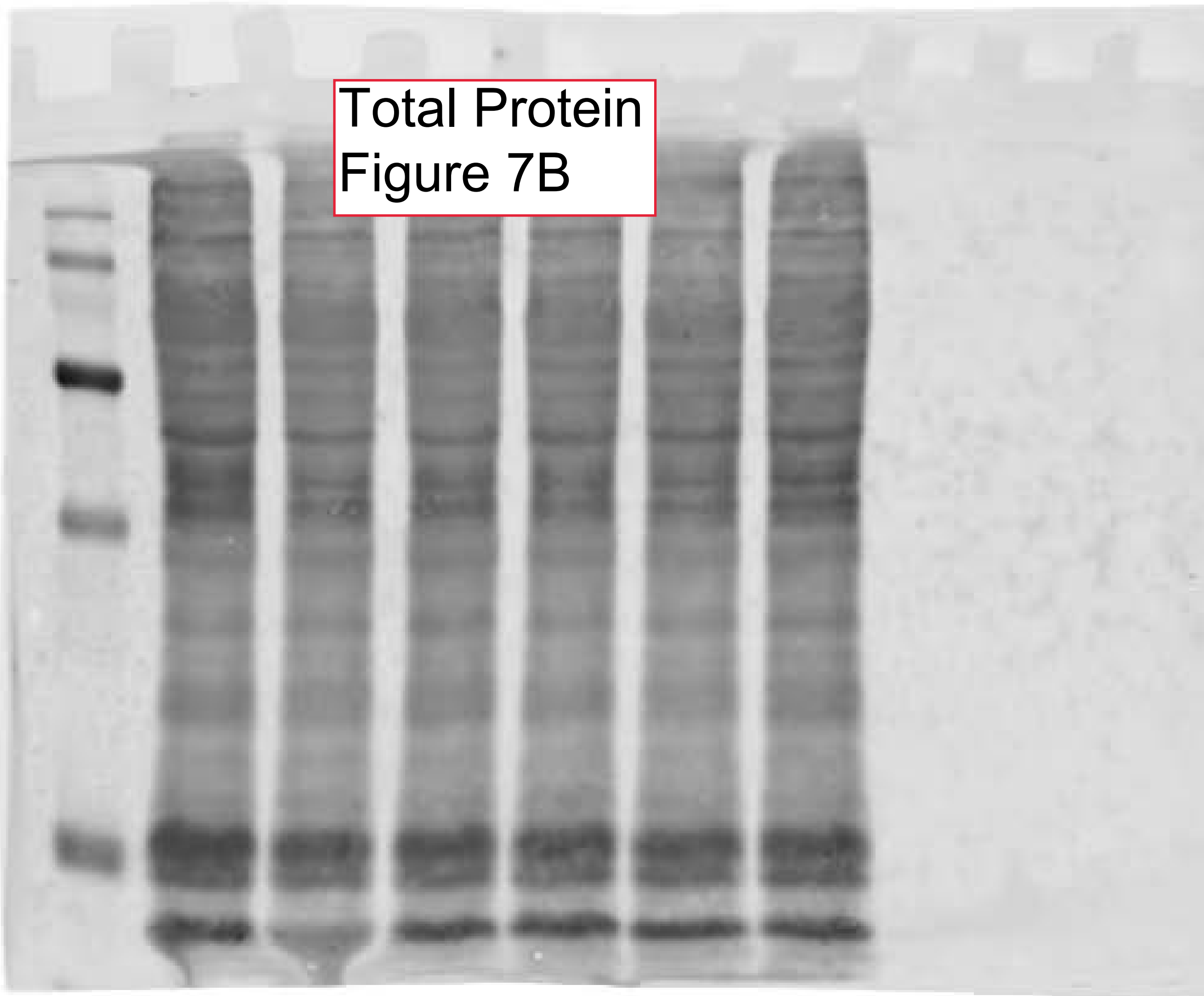
Total Protein
Figure 7B

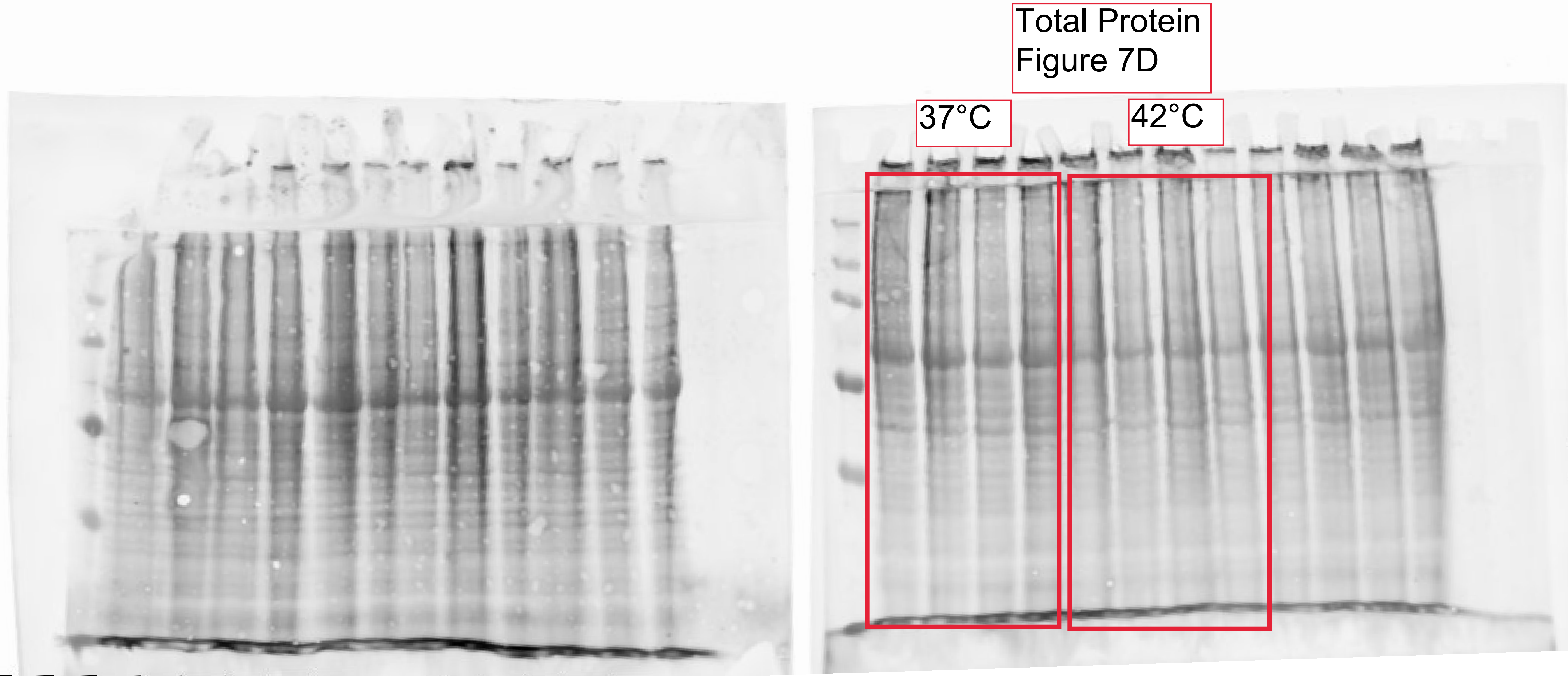
Total Protein
Figure 7D
37°C
42°C
